# WEE1 inhibitor exerts synergistic effect with KRAS G12C inhibitor via MYBL2-RRM2 axis in KRAS^G12C^-mutant lung cancer

**DOI:** 10.1038/s41419-025-07992-4

**Published:** 2025-08-30

**Authors:** Chao Zhou, Yuqing Liu, Hongyu Liu, Jun Lu, Bo Zhang, Liang Zhu, Shuyuan Wang, Huimin Lei, Baohui Han

**Affiliations:** 1 Department of Respiratory and Critical Care Medicine, Shanghai Chest Hospital, Shanghai Jiao Tong University School of Medicine, Shanghai, China; 2 Department of Pharmacology and Chemical Biology, Shanghai Jiao Tong University School of Medicine, Shanghai, China; 3 Shanghai Institute of Thoracic Oncology, Shanghai Chest Hospital, Shanghai Jiao Tong University School of Medicine, Shanghai, China

**Keywords:** Targeted therapies, Checkpoints

## Abstract

The clinical effect of KRAS G12C inhibitors (G12Ci) as monotherapy is poor, prompting the development of combination treatment strategies. Here, we demonstrate that the WEE1 kinase inhibitor (WEE1i), Adavosertib, can sensitize the effect of G12Ci through the MYBL2-RRM2 axis, which is associated with poor prognosis in lung cancer. Overexpressing the MYBL2-RRM2 axis or supplementing the products of the RRM2 enzyme, dNTPs/dNs, can partially reverse this synergistic inhibitory effect. We also observed marked effects of the combination therapy in tumor xenografts models. Collectively, these results uncover the WEE1 kinase inhibitors, some of which are available clinically, as effective enhancers for G12Ci therapy.

## Introduction

Lung cancer, as the leading cause of cancer-related deaths worldwide, has the highest incidence and mortality rates [1]. Lung adenocarcinoma (LUAD) is the most common histological subtype of non-small-cell lung cancer (NSCLC), which accounts for about 40% of lung malignancies [2]. Given the poor prognosis of LUAD, understanding its molecular mechanisms is critical for developing novel therapeutic strategies. Notably, KRAS mutations drive a significant proportion (32%) of LUAD cases [3]. Most KRAS mutations occur in codons 12 and 13, with the G12C mutation being the most common, approximately 41% of KRAS-mutant LUAD [4, 5].

For decades, KRAS has been believed to be “undruggable” due to its high affinity for guanosine triphosphate (GTP) in its active state and unique near-spherical structural characteristics, leading to several failed attempts at direct inhibition [6–10]. However, the emergence of sotorasib (AMG 510), an inhibitor for the treatment of NSCLC with KRAS G12C mutation, marked an initial breakthrough in KRAS mutation research and has greatly stimulated the development of KRAS inhibitors [11]. Two covalent inhibitors of KRAS, sotorasib (AMG 510) [11, 12] and adagrasib (MRTX 849) [13, 14], have been approved by The Food and Drug Administration (FDA) for the treatment of patients with advanced NSCLC carrying KRAS G12C mutation, and over ten follow-up drugs have entered clinical trials.

Unfortunately, clinical trials have indicated that patients with KRAS G12C mutant LUAD receiving monotherapy with sotorasib or adagrasib have not achieved satisfactory therapeutic effects [11–13, 15]. While some efficacy was observed in CodeBreaK 200 trial, the median progression-free survival (mPFS) improvement with sotorasib compared to docetaxel was only one month and did not translate into overall survival (OS) benefits [15]. Similarly, in the phase III KRYSTAL-12 trial, adagrasib achieved a mPFS of only 5.49 months, highlighting its limited clinical efficacy [16].

The transient nature of the therapeutic responses to the KRAS-G12C inhibitors observed to date has highlighted the critical need for combination strategies to achieve more durable clinical outcomes [17, 18]. Numerous approaches are being explored in both preclinical studies and clinical trials. Several lines of evidence point toward combinations with immunotherapies having particular potential [19]. The first assessment of safety and efficacy of sotorasib with anti-PD-(L)1 immunotherapy from CodeBreaK 100/101 phase 1b dose exploration showed that translating this to clinical practice has proven challenging due to major toxicities of the combination, particularly immune-mediated hepatoxicity [20]. The RAS signaling pathway has several upstream and downstream mediators, which are attractive targets for combination therapies with RAS inhibitors. Current methods to enhance therapeutic efficacy include combination therapies of sotorasib with MEK inhibitors, or other tyrosine kinase inhibitors (TKIs), though we must be mindful of potential added toxicities with the combination. Unfortunately, these slight improvements in efficacy are not sufficient to grant these combinations clinical application potential. In the cohorts with two different doses of afatinib combined with sotorasib, the objective response rates (ORRs) were 20.0% and 34.8%, respectively, and did not demonstrate significant improvement in therapeutic efficacy [21]. In a preclinical study, trametinib, a MEK inhibitor, demonstrated an ORR of only 20% when combined with sotorasib, which is not a satisfactory therapeutic effect in NSCLC [21]. The combination of platinum doublet chemotherapy with sotorasib showed improved anti-tumor activity in KRAS^G12C^-mutated NSCLC, with an ORR of 56.0%, but the rate of treatment-related adverse event (TRAEs) also increased, with 52% of Grade 3-4 TRAEs [22]. While combining chemotherapy can increase efficacy, it also correspondingly increases treatment-related adverse effects, posing challenges for actual clinical application.

Limited by the slight improvement in efficacy and the corresponding increased intolerable toxicity, the combination strategies with immunotherapy, chemotherapy, or upstream and downstream targeted drugs have not demonstrated satisfactory results or promising clinical application prospects. A highly effective and low-toxicity combination strategy must be developed to address the poor monotherapy efficacy of KRAS G12C inhibitors.

WEE1 plays a key role in cell cycle regulation, particularly in mediating the entry into mitosis. Its role is critical in normal cell cycle progression and the DNA damage response (DDR) pathways, ensuring proper repair in response to DNA damage [23, 24]. Adavosertib (AZD-1775) is the first highly potent and selective WEE1 inhibitor [25]. Extensive preclinical studies have evaluated its efficacy both as a monotherapy and in combination with other treatments. Notably, numerous studies have demonstrated the synergistic activity of WEE1 inhibitors with DNA-damaging agents, including chemotherapeutics (e.g., doxorubicin, cytarabine, methotrexate, cisplatin, clofarabine, etoposide, 5-fluorouracil) and radiotherapy, in preclinical models. The underlying mechanism involves WEE1 inhibition impairing the DDR pathway, preventing cell cycle arrest after DNA damage caused by chemotherapy or radiotherapy. Consequently, cancer cells with damaged DNA continue to proliferate, accumulating extensive DNA damage until reaching a point of irreversible collapse [24].

Preclinical studies have shown that WEE1 inhibitors, either as monotherapies or in combination with targeted agents, are effective across various tumor types harboring KRAS mutation. In mutant RAS-positive leukemia and other RAS-driven malignancies, WEE1i combined with mTOR inhibition (mTORi) represents a promising therapeutic strategy [26]. In KRAS-mutant pancreatic ductal adenocarcinoma, concurrent inhibition of WEE1 and ERK demonstrated enhanced tumor growth suppression and apoptosis compared to WEE1 inhibition alone [27]. Similarly, in KRAS-mutant lung cancers, WEE1i and mTORi exhibited synergistic antitumor effects [28]. Additionally, WEE1i combination with sorafenib restored the sensitivity of KRAS-mutant cells to this multi-target tyrosine kinase inhibitor, suggesting a potential strategy for treating KRAS-mutant NSCLC [29]. Furthermore, recent studies indicated that targeting WEE1 enhances the antitumor effect in KRAS-mutated NSCLC [30, 31].

In this study, we confirm that WEE1 inhibitors and G12C inhibitors have a robust synergistic effect. Our results demonstrate that the combination of dual inhibitors induces extensive DNA replication stress, disrupts the cell cycle, causes DNA damage, and ultimately leading to cell apoptosis. Our data suggest that the observed synergistic effect may involve the MYBL2-RRM2 axis, as evidenced by partial reversal of the inhibitory effect through MYBL2-RRM2 overexpression or RRM2 enzyme products dNTPs supplementation. These findings provide a novel, safe and effective strategy for treating KRAS G12C-mutant lung cancer through the combination of WEE1 kinase inhibitors and KRAS G12C inhibitors.

## Materials and methods

### Cell culture

Six KRAS G12C mutant human lung adenocarcinoma cell lines were involved in this study, as shown in Supplementary Table 1. Human lung cancer cell lines (NCI-H2122, NCI-H2030) were obtained from Shanghai Zhong Qiao Xin Zhou Biotechnology (China), while human lung cancer cell lines (NCI-H23, NCI-H358, Calu-1, SW1573, NCI-HCC827, NCI-H1975, NCI-H1650, A549, NCI-H460, and NCI-H1299) and human normal cell lines (HEK-293T, THP1, Jurkat, HBE, Beas2B) were obtained from the American Type Culture Collection (ATCC). H2122, H23, H358, H2030, Calu-1, SW1573, HCC827, H1975, H1650, A549, H460, H1299 and Jurkat were cultured in RPMI-1640 media supplemented with 10% fetal bovine serum and 1% penicillin-streptomycin. HEK-293T and Beas2B cells were cultured in DMEM (Dulbecco’s Modified Eagle Medium) containing 10% fetal bovine serum and 1% penicillin-streptomycin. HBE cells were cultured in specialized medium for bronchial epithelial cells. THP1 cells were cultured in RPMI-1640 media supplemented with 10% fetal bovine serum, 0.05 mM β-mercaptoethanol and 1% penicillin-streptomycin. Cells were maintained at 37 °C in a humidified atmosphere of 95% air and 5% CO2. Media was changed every 48–72 h. All cell lines were routinely authenticated via short tandem repeat (STR) DNA profiling and tested for mycoplasma contamination every 6 months using MycoBlue Mycoplasma Detector (#D101, Vazyme).

### Antibodies and reagents

The primary antibodies for western blot and immunohistochemistry used in this study are listed in Supplementary Table 2. The secondary antibody HRP-labeled Goat Anti-Rabbit IgG (H + L) was purchased from Beyotime (#A0208, China). Chemical compounds sotorasib (AMG 510, molecular formula C30H30F2N6O3), adavosertib (MK-1775, molecular formula C27H32N8O2), and ZN-c3 (azenosertib, molecular formula C29H34N8O2) were purchased from Selleck (#S8830, #S1525 China) and Aladdin (#A607901 China), respectively. dNTPs (deoxy-ribonucleoside triphosphates, containing dATP, dGTP, dTTP, dCTP) were purchased from Vazyme (#P031-01, China). dNs (deoxy-nucleosides, containing dA, dG, dT, dC) were purchased from Meilunbio (#MB3135-1, #MB3136-1, #MB3134-1, #MB3133-1, China). The human lung cancer tissue microarray (AF-LucSur2202, lung adenocarcinoma cohort) was purchased from AiFang Biological, China. The study involving the tissue microarray (TMA) was approved by the Life Sciences Ethics Committee of Changsha Yaxiang Biotechnology Co., LTD. The Ethics report is available online at yxswll.ccrl.cn using the query code 17ZK2S93GK12QX.

### Cell growth and viability assay

Cells were seeded at a density of 4000–7000 cells per well in 96-well plates. Cell growth was monitored using the IncuCyte ZOOM live cell analysis system (Essen BioScience). After 72 h of drug incubation, cell viability was determined using the Cell Counting Kit-8 (CCK-8; Dojindo) assay according to the manufacturer’s instructions.

### Colony formation assay

Cells were seeded into 12-well plates (2000 cells per well) or 96-well plates (200 cells per well) and allowed to adhere overnight. Cells were then cultured in the absence or presence of drugs for about 10 days. Remaining cells were fixed with 4% Polyformaldehyde, stained with 0.5% crystal violet, and photographed using a digital scanner after drying. For 96-well plates stained with crystal violet, 10% glacial acetic acid was added to dissolve the crystal violet, and the optical density of each well at 595 nm was measured to quantify colony formation ability.

### Drug synergistic effect

Drug synergistic effect was evaluated by the synergy scores and combination effect analysis. The average values for each concentration of single drugs or drug combinations from three biological replicates were uploaded to the Synergyfinder online tool (https://synergyfinder.org/) with viability as a readout to calculate the respective HSA synergy scores. HSA score above 10 suggests synergy. Treatment combinations will result in synergistic, additive, antagonistic effects. These effects were evaluated by a calculation of Combination Index (CI) according to the Chou-Talalay method. Data were analyzed using CompuSyn software (CompuSyn Inc.): CI = 0.85 to 0.9, slight synergism; CI = 0.7 to 0.85, moderate synergism; CI = 0.3 to 0.7, synergism; CI = 0.1 to 0.3, strong synergism; CI < 0.1, very strong synergism.

### Cell migration assay

Cells were seeded in 96-well plates and incubated at 37 °C until the bottom of the well was completely covered by a monolayer of cells. Wounds were made using a scratching tool (WoundMakerTM). Cells were cultured in the absence or presence of drugs for about 2 days. Cell growth was monitored using the IncuCyte ZOOM live cell analysis system (Essen BioScience). Photographs of wound closure were taken at the initial time point and specific time points.

### Cell cycle and apoptosis assays

Cell cycle phases and apoptosis were determined by flow cytometry analyses based on propidium iodide (PI) staining of cellular DNA content and annexin V–fluorescein isothiocyanate (FITC)/PI double staining of cell death, respectively, according to the reagent kit manufacturer’s instructions (Multi Sciences, CCS012; Multi Sciences, AT101). All samples were analyzed on a Flow Cytometer (Thermo Scientific, Attune NxT). At least 10,000 events were assessed per test. FlowJo-V10 software was used to quantify populations.

### EdU (5-ethynyl-2’ -deoxyuridine) incorporation assays

DNA synthesis was analyzed using the BeyoClick™ EdU-594 Cell Proliferation Kit (C0078, Beyotime) according to the manufacturer’s instructions. Images of the cells were captured with a fluorescence microscope, and the number of EdU-positive cells were counted using ImageJ software.

### DNA damage assay

DNA damage assay was analyzed by the γ-H2AX Immunofluorescence (C2037, Beyotime) according to the reagent kit manufacturer’s instructions. Images of the cells were captured with a fluorescence microscope, and the number of γH2AX positive cells were counted using ImageJ software.

### Flow cytometry

To measure cell cycle distribution, cells were treated with compounds for 24 h and then incubated with 10 μM EdU. After 2 h, cells were harvested by trypsinization and washed with ice-cold PBS. Cells were fixed in 4% polyformaldehyde for 15 min and then permeabilized with 0.5% Triton X-100 in PBS for 20 min at room temperature, followed by blocking with 3% BSA in PBS. The BeyoClick™ EdU-647 Cell Proliferation Kit (C0081, Beyotime) was used according to the reagent kit manufacturer’s instructions. Subsequently, DNA Damage Assay Kit by γ-H2AX Immunofluorescence (C2037, Beyotime) was used following the reagent kit manufacturer’s instructions. Finally, cells were stained with 20 mg/ml propidium iodide (PI) containing 100 mg/ml RNase A. All samples were analyzed on a Flow Cytometer (Thermo Scientific, Attune NxT). At least 10,000 events were assessed per test. FlowJo-V10 software was used to quantify populations.

### RNA sequencing (RNA-seq) analysis

H2122 cells were exposed to vehicle, sotorasib (4 μM), adavosertib (400 nM), or their combination for 24 h. Total RNA samples were extracted with an RNA extraction kit (Takara) according to the manufacturer’s manual. Libraries were sequenced on an Illumina HiSeq 4000 platform. After quality control of raw reads, the clean reads were mapped to the human genome using default parameters. Differentially expressed gene (DEG)–normalized read counts [fragments per kilobase of exon per million (FPKM)] were calculated using RSEM (v1.2.8). The Kyoto Encyclopedia of Genes and Genomes (KEGG) pathways and Gene Ontology (GO) were annotated using the KEGG pathway database (http://www.genome.jp/kegg/) and Gene Ontology Database (http://www.geneontology.org/), respectively. A false discovery rate (FDR)–corrected *P* value of 0.05 was considered significant. GSEA (Gene Set Enrichment Analysis) was performed using GSEA 4.3.0 software. IPA (Ingenuity Pathway Analysis) was performed using IPA software.

### Luciferase reporter assays

Luciferase reporter assays were achieved using firefly luciferase reporter constructs designed and synthesized by GeneChem. 1 × 10^5^ H2122, H23 cells were seeded onto 24-well plates per well and allowed to adhere overnight. The next day, the cells were co-transfected with 0.25 μg firefly luciferase reporter constructs, 0.25 μg transcription factor constructs and 10 ng pRL-SV40 Renilla luciferase reporter plasmids for 48 h. The pRL-SV40 plasmid was used to normalize the transfection efficiency. The luciferase activities were detected by luminometer (20/20 Luminometer, Promega) according to the Dual-Luciferase Reporter Assay System (E1910, Promega) technical manual. All results are representative of three independent experiments.

### RNA extraction and quantitative PCR

RNA from cells was extracted using an RNA extraction kit (RC101, Vazyme) according to the manufacturer’s instructions. The concentrations were measured with NanoDrop (Thermo Fisher Scientific), and reverse transcription was carried out using the RevertAid First Strand cDNA Synthesis Kit (Qiagen). Real-time PCR was performed in a LightCycler 480 II system (Roche) using default reaction settings. Sequences of primers for Real-Time Quantitative Reverse Transcription PCR (qRT-PCR) are shown in Supplementary Table S3.

### Gene knockdown and gene overexpression

Gene knockdown was achieved using siRNA purchased from GenePharma. Cells were plated on six-well plates at about 30% confluence and allowed to adhere overnight. Then, cells were transfected with 20 nM siRNA duplexes using Lipofectamine 3000 (Invitrogen) according to the manufacturer’s instructions. Gene knockdown was detected by Western blot or RT-qPCR analysis after 48 to 72 h of transfection. Cells transfected with mock siRNA duplexes were used as controls. Target sequences for siRNAs are shown in Supplementary Table S4. Gene overexpression was achieved using MYBL2 and RRM2 plasmids designed and synthesized by GeneChem. Cells were plated on six-well plates and allowed to adhere overnight. The cells were transfected with 2.5 μg plasmid at approximately 40–50% confluence using Lipofectamine 3000 reagent (Invitrogen) according to the manufacturer’s instructions. After 48 h of transfection, the cells were harvested and used for further experiments. Gene overexpression was detected by Western blot after 48 h of transfection.

### Western blot analysis

Cells were washed with cold phosphate-buffered saline (PBS) and lysed using RIPA buffer (Beyotime) with protease inhibitor and phosphatase inhibitor cocktail (Beyotime). The protein concentrations were determined using a bicinchoninic acid (BCA) Protein Assay Kit (Thermo Fisher Scientific). Approximately 10 to 30 µg of protein samples were loaded in SDS-polyacrylamide gel electrophoresis (SDS-PAGE) for separation and run for approximately 1 h at 150 V, followed by transfer from the gel to a nitrocellulose membrane at 400 mA for 50 min. Membranes were blocked with 5% nonfat milk in 1× tris-buffered saline–Tween 20 for 1 h at room temperature and incubated with diluted primary antibodies at 4 °C with gentle shaking overnight. After incubation with horseradish peroxidase–conjugated anti-rabbit immunoglobulin G (YEASEN) or anti-mouse IgG antibodies (YEASEN), the immunoblots were subjected to electrochemiluminescence (Thermo Fisher Scientific) and scanned using an Odyssey FC imaging system (LI-COR Biosciences).

### Xenograft studies

For tumorigenesis, 1 × 10^7^ H2122 cells (resuspended in 100 μL serum-free 1640 media) were subcutaneously administered into the bilateral flanks of six-week-old female BALB/c nu/nu athymic mice. Subcutaneous local tumors were measured using a digital caliper every 3 days, and tumor volumes were calculated using the formula: volume = length × width^2^ × 0.5. For treatment, mice were randomized into groups (*n* = 5 mice per treatment group) with similar mean tumor volumes, reaching approximately 270 mm^3^. Sotorasib was dissolved in 5% DMSO + 1% Tween 80 + ddH_2_O and administered by oral gavage once a day at 100 mg/kg. Adavosertib was dissolved in 4% DMSO + 1% Tween 80 + ddH_2_O and administered by oral gavage once a day at 40 mg/kg. Mice received one of the following treatments: 1) vehicle; 2) sotorasib 100 mg/day/kg; 3) adavosertib 40 mg/day/kg; or 4) a combination of sotorasib and adavosertib. Tumors were harvested for weight measurement, H&E staining, and Immunohistochemistry (IHC) staining. Mice were housed in ventilated cages in an animal barrier facility at the Shanghai Jiao Tong University School of Medicine (Shanghai, China). All mice were maintained in a specific pathogen-free room at 22° to 26 °C with a 12/12-hour light/dark schedule and fed with sterile pellet food and water ad libitum. All procedures and experiments involving animal studies were evaluated and approved by the Institutional Animal Care and Use Committee (IACUC) and carried out in accordance with the Animal Care and Use Rules of Shanghai Jiao Tong University School of Medicine.

### Histology, IHC and TUNEL staining

Tumors were fixed with 4% paraformaldehyde overnight, embedded in paraffin, and then cut into 4 μm-thick sections for H&E staining. For IHC staining, Ki67, MYBL2, RRM2, and γH2AX protein levels in the tumor samples harvested from mice were assessed by IHC using anti-Ki67 (1:1600 dilution, ABclonal), anti-MYBL2 (1:1000 dilution, Proteintech), anti-RRM2 (1:1200 dilution, ABclonal), and anti-γH2AX (1:800 dilution, ABclonal). For IHC staining in human LUAD tissue, MYBL2 and RRM2 protein levels in the tumor and normal lung samples were assessed by IHC using anti-MYBL2 (1:400 dilution, Proteintech) and anti-RRM2 (1:400 dilution, ABclonal). Briefly, after de-paraffinization, endogenous peroxidase activity was blocked by pre-treatment with 3% H_2_O_2_. The slides were incubated with normal goat serum for 30 min at RT to block non-specific signals, then incubated with the primary antibody overnight at 4 °C. The slides were then incubated with polymer horseradish peroxidase-labeled secondary antibodies for 45 min at RT, followed by 3,3-Diaminobenzidine (DAB) treatment (0.05 g DAB and 100 ml 30% H_2_O_2_ in 100 mL PBS) for 5 min. The nucleus was stained with hematoxylin for 2 minutes and observed under a microscope. The terminal transferase mediated d-UTP nick end-labeling kit (TUNEL) staining were used to detect the tumor cell apoptosis in vivo. The TUNEL assay was performed according to the manufacturer’s instructions (TUNEL BrightRed Apoptosis Detection Kit, #A113, Vazyme). Apoptosis index (AI) (%) = the number of positive (TMR red) cells/all cells (DAPI) in field of view.

### Public database analysis

The expression data and clinical data of TCGA LUAD were retrieved from TCGA LUAD datasets (http://cancergenome.nih.gov/). Expression plots of TCGA datasets were performed using GEPIA 2 (http://gepia2.cancer-pku.cn/). The expression data and clinical data of GSE30219 and GSE31210 were retrieved from GEO datasets (https://www.ncbi.nlm.nih.gov/geo/). Patients from GSE31210 were stratified according to high versus low expression (cutoff: median) of MYBL2 or RRM2 in their tumors. The expression data and clinical data of tissue microarray were obtained from AiFang Biological. Patients from tissue microarray were stratified according to high versus low expression (cutoff: median) of MYBL2 or RRM2 in their tumor tissues. The clinical characteristics of patients in TMA are listed in Supplementary Table S5.

### Statistical analysis

The experimental data were collected in three separate experiments and expressed as means ± SD (standard deviation). Statistical analysis was performed using two-tailed Student’s *t* test or analysis of variance (ANOVA) test. The survival curves were generated using the Kaplan-Meier method for prognostic analysis, and the significance of differences was determined by log-rank tests. For the sake of presentation, *p* values > 0.05 were denoted as ‘ns’, *p* values < 0.05 were denoted as ‘*’, *p* values < 0.01 were denoted as ‘**’, *p* values < 0.001 were denoted as ‘***’, and *p* values < 0.0001 were denoted as ‘****’. All analyses were performed using GraphPad Prism V.9 software, and a *p* value < 0.05 was considered statistically significant.

## Results

### WEE1 inhibitor exhibits synergistic effects with KRAS G12C inhibitor

**Fig. 1:**
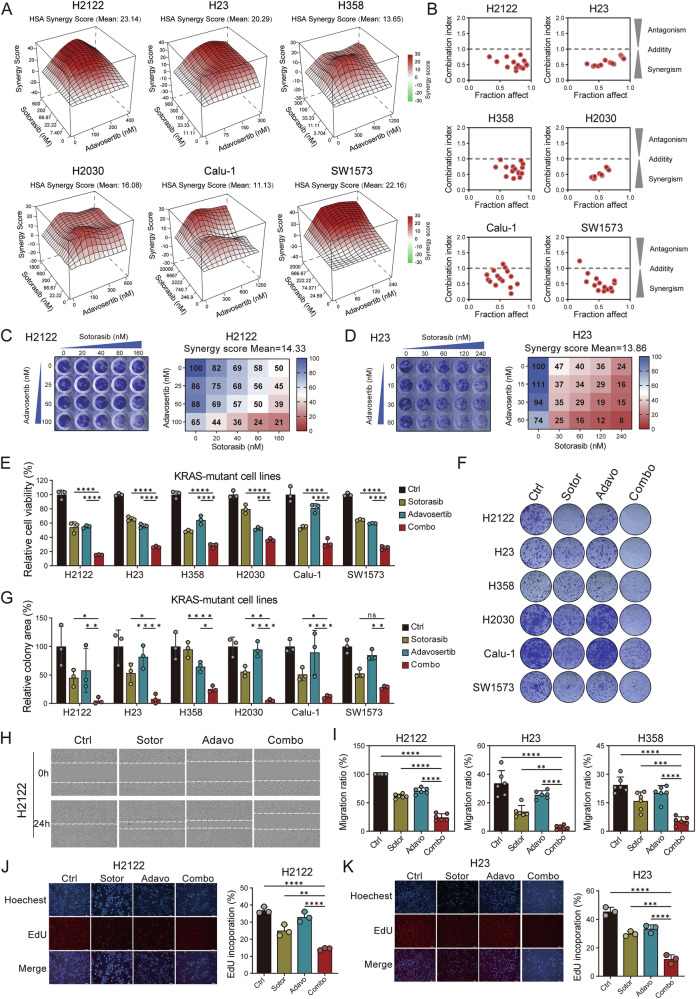
Combination of sotorasib and adavosertib is synergistic. **A** HSA synergy maps for the combination of sotorasib and adavosertib in H2122, H23, H358, H2030, Calu-1, and SW1573 cells. Synergyfinder.org web tool was used to calculate the synergy scores. A score above 10 indicates drug synergy. **B** Synergistic effect of sotorasib and adavosertib calculated using CI values. CI value was calculated as described in Materials and Methods. **C, D** Colony formation assay and HSA synergy score of H2122 (**C**) and H23 (**D**) cell lines treated with gradient concentrations of sotorasib and adavosertib for 10-14 days. **E** Cell viability of H2122, H23, H358, H2030, Calu-1, and SW1573 cells under untreated, sotorasib, adavosertib or combination treatment conditions for 72 h. The data were normalized to untreated controls. *n* = 3 per group. **F** Representative images of colony formation in H2122, H23, H358, H2030, Calu-1, and SW1573 cells under treatment for 10-14 days. **G** The relative colony area data in H2122, H23, H358, H2030, Calu-1, and SW1573 cells under treatment were calculated for 10-14 days. The data were normalized to untreated controls. *n* = 3 per group. **H** Representative images of cell scratch assay in H2122 under treatment for 24 h. **I** Migration ratio to evaluate migration ability in H2122, H23 and H358 for 24–48 h. **J, K** Representative images and proportion of EdU incorporation assay in H2122 (**J**) and H23 (**K**) under treatment for 48 h. Data are mean ± SD of three independent replicates.

Considering the strong synergistic effects of WEE1 kinase inhibitors with other downstream target inhibitors in KRAS-mutated lung cancer [26–31], we hypothesize that WEE1 inhibitors can also exert synergistic effects with KRAS G12C targeted inhibitors. We utilized six KRAS G12C-mutated lung cancer cell lines in our study: H2122, H23, H358, H2030, Calu-1, and SW1573. Adavosertib (AZD-1775) is the first highly potent and selective WEE1 inhibitor and numerous preclinical studies have evaluated its efficacy as a single agent and in combination approaches [24]. Sotorasib (AMG 510) is the first inhibitor approved by the FDA for the treatment of KRAS G12C-mutated NSCLC [12]. By assessing the drug sensitivity curves for the KRAS G12C inhibitor (Sotorasib) and the WEE1 inhibitor (Adavosertib), we determined the gradient drug concentrations to test cell viability at the indicated concentrations (Supplementary Fig. S1A–B). Meanwhile, Given that the clinical advancement of adavosertib has been constrained by dose-limiting toxicities (particularly hematologic adverse effects) [32–34], we systematically evaluated its therapeutic window across multiple cellular models including normal lung epithelial cells, monocytes, T cells, and lung cancer cell lines harboring wild-type KRAS or non-G12C KRAS mutations (Supplementary Fig. S1C). By assessing cell viability in different concentration combinations of the two drugs, we analyzed the synergistic effects of the drugs using cell viability assays (Supplementary Fig. S1D). The synergy effects were evaluated using synergy scores from Synergyfinder and the combination index (CI) values from CompuSyn software to minimize errors from a single assessment method. Synergistic effects were considered if the synergy score was greater than 10 and the CI was less than 1. Consequently, the mean synergy scores of adavosertib combined with sotorasib were all greater than 10, and most CI values were less than 1 for the six KRAS G12C mutant lung cancer cell lines, indicating a synergistic effect in both sotorasib-sensitive H358 and sotorasib-resistant H2122, H23, H2030, Calu-1, and SW1573 cell lines (Fig. 1A, B). We used colony formation assay to reflect long-term cell growth capacity. In H2122 and H23 cell lines, the ability of cells to form colonies under different combinations of the two drugs at varying concentrations also demonstrated a synergistic effect (Fig. 1C, D).

Recently, ZN-c3 (azenosertib), which has higher WEE1 selectivity, showed improved safety results compared to those of adavosertib [35] (Supplementary Fig. S1E). Similar synergistic effects were observed in treatment combined with ZN-c3, as in KRAS-G12C mutant cell lines treated with the combination of sotorasib and adavosertib (Supplementary Fig. S1F, G). Furthermore, we examined the long-term cell growth by conducting a colony formation assay to examine the combined effects of G12C inhibitor and WEE1 inhibitor. We found that, regardless of whether it was adavosertib or ZN-c3, the combination with sotorasib also showed significant synergistic effects in the long-term cell viability assays in the H2122 and H23 cell lines (Fig. 1C, D and Supplementary Fig. S1H–J). Overall, these experiments revealed robust synergistic interactions with WEE1 inhibitors and sotorasib in all KRAS-G12C mutant LUAD cell lines.

Given the synergistic effects observed in cell viability and growth, we investigated whether the combination might also have synergistic impacts on other cellular biological characteristics. In six KRAS G12C-mutated cell lines, the combination of sotorasib and adavosertib significantly inhibited cell viability and reduced long-term survival at the indicated concentrations (Fig. 1E–G). Cell scratch assay was used to study the cells’ migration ability (Fig. 1H and Supplementary Fig. S1K and L), and the combination treatment significantly inhibited the migration ability of H2122, H23, and H358 cells (Fig. 1I). We employed the EdU incorporation assay to explore cell proliferation in H2122 and H23 cells. The results showed that the combination significantly reduced the proportion of EdU-incorporating cells, indicating a significant inhibition of proliferative capacity (Fig. 1J, K).

In summary, these KRAS G12C-mutated lung cancer cell lines exhibited marked sensitivity to the combination of sotorasib and adavosertib, demonstrating a strong synergistic effect.

### Combination of G12Ci and WEE1i induces cell cycle perturbation, replication stress, and DNA damage, leading to apoptosis

WEE1 kinase is involved in the regulation of S and G2/M phase cell cycle checkpoints and acts as the gatekeeper of the G2/M cell cycle checkpoint, allowing DNA repair before mitotic entry [23]. The recognized function of WEE1 inhibitors is to permit mitosis without repairing DNA damage, leading to replication stress and the accumulation of DNA damage response (DDR), ultimately resulting in apoptosis [24]. Considering the critical role of WEE1 kinase in the cell cycle and apoptosis, we first investigated the impact of combining WEE1 inhibitors and G12C inhibitors on the cell cycle, apoptosis and DNA damage.

**Fig. 2:**
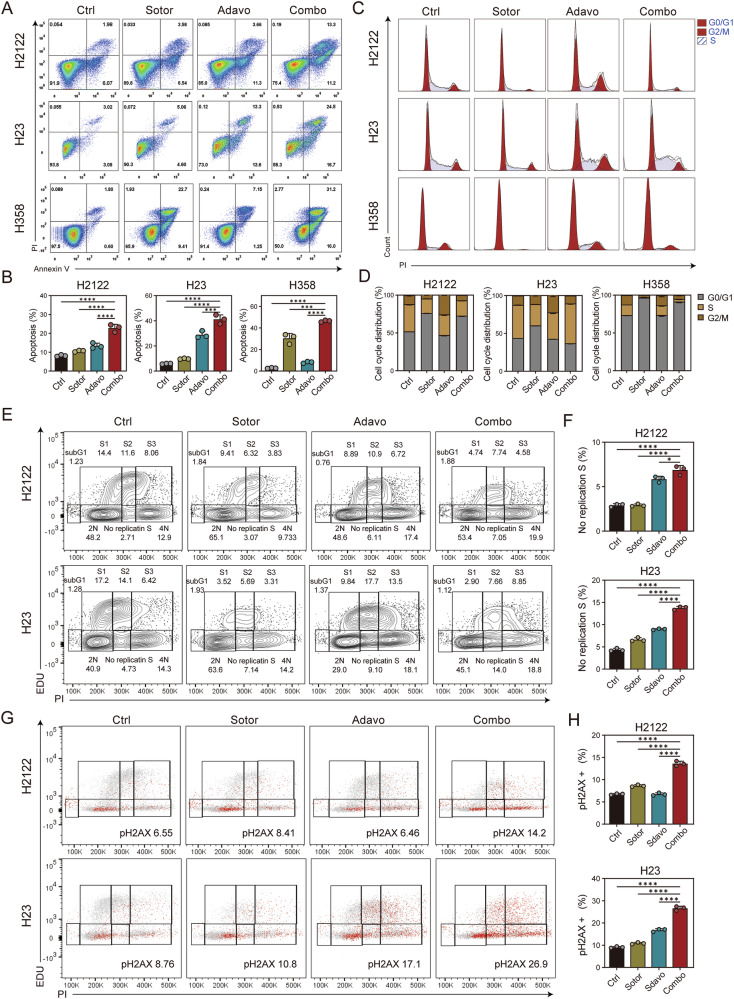
Combination of G12Ci and WEE1i induces cell cycle perturbation, replication stress, and DNA damage, leading to apoptosis. **A** Representative images of cell apoptosis assay in H2122, H23 and H358 cells under untreated, sotorasib, adavosertib or combination treatment for 48 h. **B** Cell apoptosis proportion of cell apoptosis assay in H2122, H23 and H358 cells under treatment for 48 hours. *n* = 3 per group. **C** Representative images of cell cycle assay in H2122, H23 and H358 cells under treatment for 48 h. The cell cycle was divided into the G0/G1, S and G2/M phases. **D** Cell cycle distribution in H2122, H23 and H358 cells under treatment for 48 h. *n* = 3 per group. **E** Representative images of cell cycle distribution in H2122 and H23 cells under treatment for 48 h. The cell cycle was divided into the 2 N, 4 N, S1, S2, S3, no replication S, and subG1 phases by PI and EdU. **F** Proportion of no replication S phase in H2122 and H23 cells under treatment for 48 h. *n* = 3 per group. **G** Representative images and proportion of γH2AX in H2122 and H23 cells under treatment for 48 h using flow cytometry. **H** The proportion of γH2AX positive cells in H2122 and H23 cells under treatment for 48 h using flow cytometry. *n* = 3 per group. Data are mean ± SD of three independent replicates.

By detecting the proportion of early and late apoptosis in H2122 and H23 cells, we found that the combination therapy significantly increased the proportion of apoptotic cells, illustrating the synergistic effect of the combination (Fig. 2A, B). We also assessed the expression of γH2AX, a marker of DNA damage. The results indicated that the combination therapy increased the proportion of γH2AX-positive cells, signifying an escalation in DNA damage (Supplementary Fig. S2A, B). However, in the cell cycle experiments, the changes in cycle distribution did not show a consistent and regular pattern between the combination and control groups (Fig. 2C, D). After treating H2122, H23, and H358 cells with sotorasib, adavosertib, or a combination of both for 48 h, we measured the propidium iodide (PI) content by flow cytometry to distinguish between the G0/G1, S, and G2/M phases. We found that sotorasib treatment drastically increased the proportion of cells in the G0/G1 phase, indicating its ability to prevent cells from undergoing mitosis, consistent with previous findings [36]. Adavosertib treatment strongly elevated the proportion of cells in the G2/M and S phases, which aligned with the known effects of WEE1 inhibitors and previous research results [30]. Therefore, the combination treatment did not exhibit regular changes in the proportions of cells in the G0/G1, S, and G2/M phases. Consistent with the results from 48 h of treatment, the changes in the cell cycle after 24 h also did not show observable differences in the cell cycle distributions with the combination therapy (Supplementary Fig. S2C, D). In summary, this suggests that the combination therapy exerts a synergistic effect on apoptosis, but this cannot be explained by changes in the cell cycle.

Furthermore, we detected the expression of proteins related to the cell cycle and apoptosis by western blot analysis (Supplementary Fig. S2E). Regarding cell cycle-related proteins, the combination treatment synergistically inhibited the expression of p-CDK1 and Cyclin E2, proteins associated with cell activity. In terms of apoptosis-related protein expression, cleaved caspase 3 and 7 significantly increased with the combination treatment, indicating a significant increase in cellular apoptosis. Overall, these results demonstrated that the combination therapy had a strong impact on the cell cycle and apoptosis, characterized by significant increases in apoptosis-related proteins and changes in cell cycle-related proteins.

Given the limitations of using PI to distinguish the proportions of the cell cycle, we tracked cell cycle progression and DNA replication using PI and 5-Ethynyl-2′-deoxyuridine (EdU), and DNA damage was assessed using γH2AX labeling flow cytometry. After 24 h of treatment, flow cytometry was used to analyze the proportions and distribution of EdU, PI, and γH2AX. The cell cycle was divided into the 2 N, 4 N, S1, S2, S3, no replication S, and subG1 phases (Fig. 2E and Supplementary Fig. S3A–C). The no replication S phase exhibited DNA content between 2 N and 4 N but did not incorporate the synthetic nucleoside EdU, indicating DNA replication stress [37, 38]. Cells in this situation exhibit DNA damage and mitotic catastrophe, as revealed by γH2AX expression, which also indicates a high level of DNA damage in the no replication S phase. In our cell cycle detection, we observed that combination treatment forced H2122 and H23 cells to accumulate in the no replication S phase, indicating replication stress (Fig. 2E, F).

Simultaneously, by detecting the proportion of γH2AX, we found that the synergistic effect was also reflected in DNA damage. The combination therapy significantly increased the proportion of cells with DNA damage, reflecting mitotic catastrophe during DNA replication (Fig. 2G, H). By statistically analyzing the γH2AX proportions in each cell cycle phase, it was observed that DNA damage primarily occurs during the DNA replication process (Supplementary Fig. S3E, F). The increased DNA damage due to the combination therapy was mainly manifested in the 4 N, S2, S3, and no replication S phases, confirming that the synergistic effect of WEE1 inhibitors and sotorasib on DNA damage is triggered by DNA replication stress.

In summary, we demonstrated that WEE1 inhibitors and sotorasib synergistically promote cell apoptosis. This outcome was achieved by inducing DNA replication stress, leading to the accumulation of DNA damage, and ultimately resulting in cell cycle perturbation and mitotic catastrophe.

### The synergistic effect of WEE1i and G12Ci may depend on the MYBL2-RRM2 pathway

To elucidate the mechanism by which the combination of sotorasib and adavosertib exerts a synergistic effect, we employed RNA sequencing (RNA-seq) to investigate the underlying molecular mechanisms. H2122 cells were treated with 4 μM sotorasib, 400 nM adavosertib, or their combination for 24 h, after which they were collected for RNA-seq analysis. We conducted Gene Ontology (GO), Kyoto Encyclopedia of Genes and GenomesGene Ontology (KEGG), Gene Set Enrichment Analysis (GSEA), and Ingenuity Pathway Analysis (IPA) on the sequencing results to uncover the molecular mechanisms and key pathways involved in the combination treatment (Supplementary Fig. S4A, B).

**Fig. 3:**
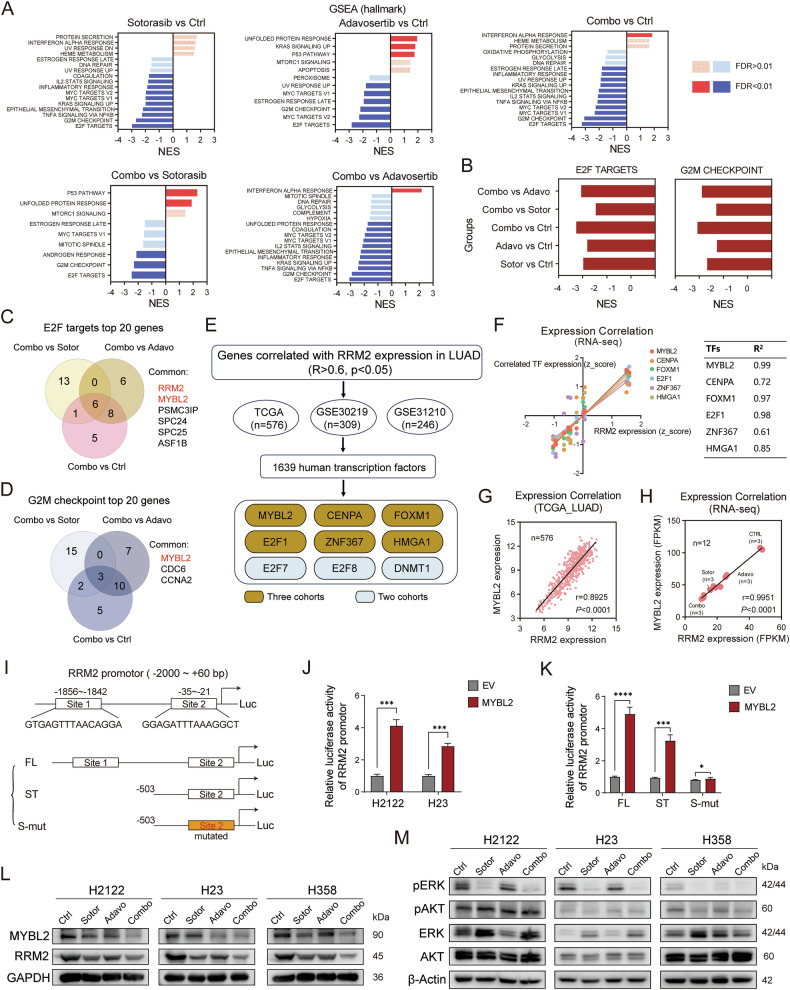
The synergistic effect of WEE1i and G12Ci may depend on the MYBL2-RRM2 pathway. **A** GSEA analysis comparing sotorasib, adavosertib, and combination groups to the control group, as well as comparing the combination group to sotorasib and adavosertib groups. **B** NES values of E2F targets and G2M checkpoint pathways in GSEA analysis, with pairwise comparisons among the four treatment groups. **C** Venn diagram of the top 20 genes in E2F targets pathway comparing the combination group to sotorasib group, adavosertib group and control group. **D** Venn diagram of the top 20 genes in G2M checkpoint pathway comparing the combination group to sotorasib group, adavosertib group and control group. **E** Flowchart illustrating the identification of RRM2 transcription factors using TCGA LUAD and GSE31210 and GSE30219 datasets. **F** The correlation between MYBL2, CENPA, FOXM1, E2F1, ZNF367, and HMGA1 with RRM2 in RNA-seq data. **G, H** The correlation between MYBL2 and RRM2 in TCGA LUAD (**G**) and our RNA-seq (**H**). **I** The schematic diagram for two MYBL2 binding motifs on RRM2 promoter (FL) and related truncated (ST) and site-mutated promoter (S-mut) constructs. **J** Relative luciferase reporter activity of RRM2 promoter (−2000 ~ + 60) while co-transfected with MYBL2 expression plasmid for 48 h in H2122 and H23 cells. **K** Relative luciferase reporter activity of the truncated and site-mutated RRM2 promoters while co-transfected with MYBL2 expression plasmid for 48 h in H2122 cells. **L** The expression of MYBL2 and RRM2 proteins in H2122, H23 and H358 cells under control, sotorasib, adavosertib or combination treatment for 24 h. **M** The expression of phosphorylation and total levels of ERK and AKT proteins in H2122, H23 and H358 cells under control, sotorasib, adavosertib or combination treatment for 24 h.

GO and KEGG analysis indicated that the genes upregulated in the drug-treated group are concentrated in the p53 signaling pathway, while the downregulated genes in the drug-treated group are concentrated in cell division, cell cycle, and DNA replication (Supplementary Fig. S4C–F). We performed GSEA analysis on the expression data to investigate global gene and key pathway changes. GSEA analysis revealed that both sotorasib and adavosertib caused significant downregulation of the E2F targets and G2M checkpoint pathways. Furthermore, the combination treatment group showed a more pronounced inhibition of these two pathways compared to the control or single-agent groups (Fig. 3A, B and Supplementary Fig. S5A, B). IPA canonical pathway analysis also revealed that the cell cycle checkpoint, activation of the pre-replicative complex, and cell cycle control of chromosomal replication are downregulated pathways, which is consistent with our GSEA analysis findings for the E2F targets and G2M checkpoint pathways (Supplementary Fig. S5C). These findings demonstrate that E2F targets and the G2M checkpoint are key signaling pathways through which the combination therapy exerts its synergistic effects.

We identified the E2F targets and G2M checkpoint pathways as the most critical for our analysis. Consequently, we focused on the top 10 genes within these two pathways. In pairwise comparisons among the four treatment groups, MYBL2 and RRM2 consistently emerged as the top two upregulated genes in the E2F pathway and RRM2 was identified as the most significantly upregulated gene in the G2M pathway (Fig. 3C, D and Supplementary Fig. S5B). Based on these findings, we hypothesize that MYBL2 and RRM2 are key genes contributing to the synergistic effects observed. It is noteworthy that MYBL2 functions as a transcription factor, while RRM2 is an enzymatic protein. This finding led us to explore the relationship between MYBL2 and RRM2.

Ribonucleotide reductase M2 (RRM2) is a small subunit in ribonucleotide reductases, which participate in nucleotide metabolism and catalyze the conversion of nucleotides to deoxynucleotides, maintaining the dNTPs pools for DNA biosynthesis, repair, and replication [39]. MYB Proto-Oncogene Like 2 (MYBL2), a member of the MYB transcription factor family, is a crucial regulatory factor that mediates cell cycle progression, cell survival, and differentiation [40]. Interestingly, a study has indicated that MYBL2 directly binds to the RRM2 promoter and promoted its transcription in colorectal cancer cells [41].

Therefore, we validated this conclusion in lung adenocarcinoma databases. We utilized three lung adenocarcinoma databases, TCGA LUAD data, GSE31210 and GSE30219, alongside the human transcription factors (TFs) database to identify the transcription factors of RRM2. The results revealed that MYBL2, CENPA, FOXM1, E2F1, ZNF367, and HMGA1 are the most relevant upstream transcription factors in lung adenocarcinoma (Fig. 3E). In our RNA-seq analysis, we examined the correlation between six transcription factors and the downstream gene RRM2. Our results revealed that MYBL2 is the most highly correlated transcription factor with RRM2 (Fig. 3F). We calculated the correlation between MYBL2 and RRM2 in four data sets, including TCGA LUAD, our RNA-seq, GSE31210, and GSE30219 (Fig. 3G, H and Supplementary Fig. S5D, E). All showed a very strong correlation between MYBL2 and RRM2, indicating that MYBL2 probably functions as a transcription factor of RRM2 in lung adenocarcinoma.

Previous studies have demonstrated that MYBL2 directly transcriptionally regulates RRM2 expression in colorectal cancer cell lines [41]. To validate MYBL2-mediated transcriptional regulation of RRM2 in KRAS G12C mutant lung cancer, we identified two putative MYBL2-binding motifs within the predicted RRM2 promoter region (-2000 to +60). Based on this prediction, we generated truncated and site-mutated promoter constructs (Fig. 3I). Dual-luciferase reporter assays in lung cancer cell lines (H2122 and H23) demonstrated that MYBL2 overexpression significantly enhanced RRM2 promoter activity (Fig. 3J). Truncated RRM2 promoters (with deletion of site 1) and site-mutated promoters (with both sites 1 and 2 deleted) showed abrogation of MYBL2-induced reporter activity (Fig. 3K). These results conclusively establish that MYBL2 activates RRM2 transcription through directly binding to its promoter in KRAS G12C mutant lung cancer cells.

In our study, RNA sequencing results indicated that the MYBL2-RRM2 axis may play a pivotal role in exerting a synergistic effect. To further explore this, here we verified the impact of the combination of sotorasib and adavosertib on the expression of the MYBL2-RRM2 axis. H2122, H23, and H358 cells were treated with either sotorasib, adavosertib, or a combination of both. Reverse transcription PCR (RT-PCR) experiment results demonstrated that the mRNA expression levels of MYBL2 and RRM2 were the lowest in the combination treatment group, indicating that the combination treatment inhibited the MYBL2-RRM2 axis (Supplementary Fig. S5F). Western blot experiments further verified the protein expression levels of MYBL2 and RRM2, showing that the combination therapy significantly reduced the protein expression of MYBL2 and RRM2 in three cell lines, which is consistent with the previous RNA-seq results (Fig. 3L). Additionally, as the drug concentration gradient increases, the expression levels of MYBL2 and RRM2 are correspondingly inhibited. The degree of inhibition of the MYBL2-RRM2 axis is related to the concentration of the drug (Supplementary Fig. S5G). The conclusion that the combined drug administration synergistically inhibits the MYBL2-RRM2 axis is also supported by results obtained with another WEE1 inhibitor, ZN-c3 (Supplementary Fig. S5H). Notably, we examined the phosphorylation levels of ERK and AKT in the downstream signaling pathways of KRAS. It was found that the combination therapy did not significantly reduce the expression of p-ERK and p-AKT more than Sotorasib alone (Fig. 3M). This also demonstrates that the synergistic effect of sotorasib and adavosertib is independent of the KRAS downstream signaling pathways. These fundings suggest that the MYBL2-RRM2 axis is synergistically inhibited by the combination of G12Ci and WEE1i.

In summary, our sequencing results revealed that the E2F targets and G2M checkpoint pathways are the key mechanisms through which sotorasib and adavosertib exert their synergistic effects. Among these, the MYBL2-RRM2 axis is the most relevant gene pathway. Our sequencing results and experimental validation suggest that the combination therapy of G12Ci and WEE1i inhibits the MYBL2-RRM2 axis, and the synergistic effect may be mediated through the MYBL2-RRM2 axis.

### The MYBL2-RRM2 axis is associated with poor prognosis in lung adenocarcinoma

**Fig. 4:**
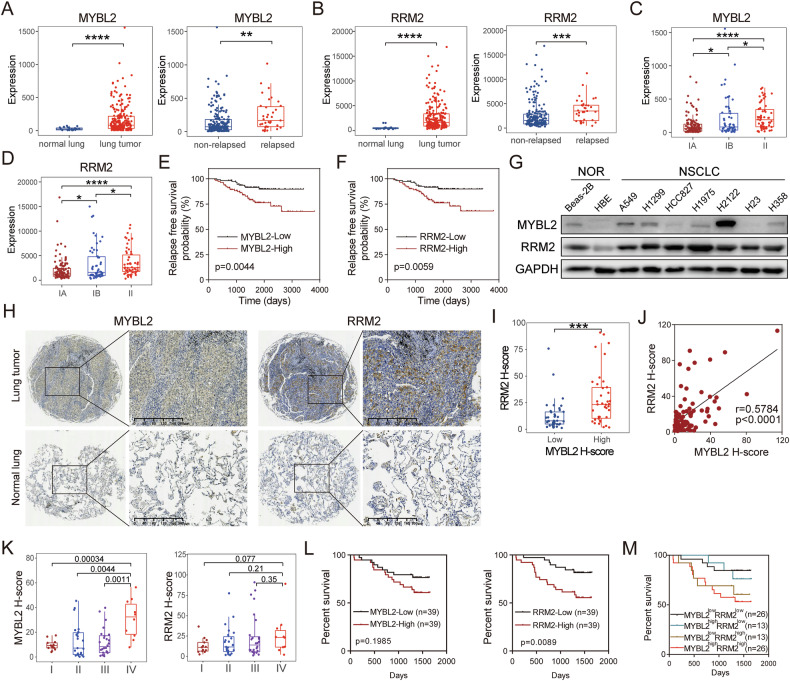
The MYBL2-RRM2 axis is associated with poor prognosis in lung adenocarcinoma. **A, B** Comparison of MYBL2 (**A**) and RRM2 (**B**) expression in normal lung tissue and early-stage lung adenocarcinoma tumors or in tumor tissues from non-relapsed and relapsed lung cancer patients in the GSE31210 dataset. **C, D** Comparison of MYBL2 (**C**) and RRM2 (**D**) expression at different stages in the GSE31210 dataset. **E, F** Relapse free survival probability of two groups classified by MYBL2 (**E**) or RRM2 (**F**) median expression levels in the GSE31210 dataset. **G** The expression of MYBL2 and RRM2 proteins in normal lung epithelial cells (NOR) and NSCLC cells (NSCLC). **H** Representative IHC images of MYBL2 and RRM2 expression in LUAD tissue microarray. **I** RRM2 expression levels in high and low expression groups based on the median value of MYBL2. **J** The correlation between MYBL2 and RRM2 IHC H score in LUAD tissue microarray. **K** Comparison of MYBL2 and RRM2 expression at different stages in the LUAD tissue microarray. **L** Percent survival probability of two groups classified by MYBL2 or RRM2 median expression levels in the LUAD tissue microarray. **M** Percent survival probability of four patient groups classified by the median expression levels of MYBL2 or RRM2 in the LUAD tissue microarray.

To evaluate the clinical significance of MYBL2-RRM2 axis in lung cancer, we first observed that MYBL2 and RRM2 are highly expressed in cancer tissues compared to adjacent normal tissues across multiple cancer types in TCGA (Supplementary Fig. S6A). This finding suggests that the MYBL2-RRM2 axis may be associated with tumor malignancy. Then, we analyzed specimens from a cohort of patients with early-stage LUAD and normal lung tissues using the GSE31210 dataset from the Gene Expression Omnibus (GEO) database. Patients with early-stage LUAD were further divided into relapse and non-relapse subgroups based on follow-up observations. Notably, MYBL2 and RRM2 showed significant overexpression in LUAD tumors compared to adjacent normal lung tissues, consistent with the findings from the TCGA dataset. Furthermore, their expression was higher in tumors from relapsed LUAD patients than in those from the non-relapsed group (Fig. 4A, B). Additionally, the expression levels of MYBL2 and RRM2 were indicative of a higher stage in LUAD (Fig. 4C, D). The expression of MYBL2 and RRM2 also showed an inverse correlation with relapse-free survival (RFS) time in LUAD patients (Fig. 4E).

We also utilized two normal lung epithelial cell lines and seven lung cancer cell lines, and found that in lung cancer cell lines, the expression of MYBL2 and RRM2 is higher, which is consistent with the results from clinical samples (Fig. 4G). We employed a tissue microarray (TMA) containing 78 lung adenocarcinoma samples with complete clinical information and performed immunohistochemical (IHC) staining of MYBL2 and RRM2. The results indicated that the expression levels of MYBL2 and RRM2 were significantly higher in cancer tissues compared to adjacent non-cancerous tissues (Fig. 4H). Moreover, the correlation between the expression of MYBL2 and RRM2 was consistent with previous sequencing data from TCGA and GEO databases, showing a significant correlation (Fig. 4I, J). Clinical staging revealed a positive correlation between higher stages of lung adenocarcinoma and increased staining intensity of MYBL2 and RRM2 (Fig. 4K). We categorized the expression of MYBL2 and RRM2 into high and low expression groups based on the median H-score and conducted a survival analysis. The findings demonstrated that high expression levels of MYBL2 and RRM2 were associated with shorter survival times in lung adenocarcinoma patients. Notably, the combined high expression of MYBL2 and RRM2 was associated with the poorest prognosis (Fig. 4L, M).

Here, by utilizing bioinformatics, lung cancer cells, and tissue microarray from lung adenocarcinoma, we examined the clinical significance of the MYBL2-RRM2 axis in lung adenocarcinoma. Our findings indicate that the MYBL2-RRM2 axis is associated with poor prognosis in lung adenocarcinoma.

### Overexpression of MYBL2 or RRM2 partially counteracts the synergistic inhibition

**Fig. 5:**
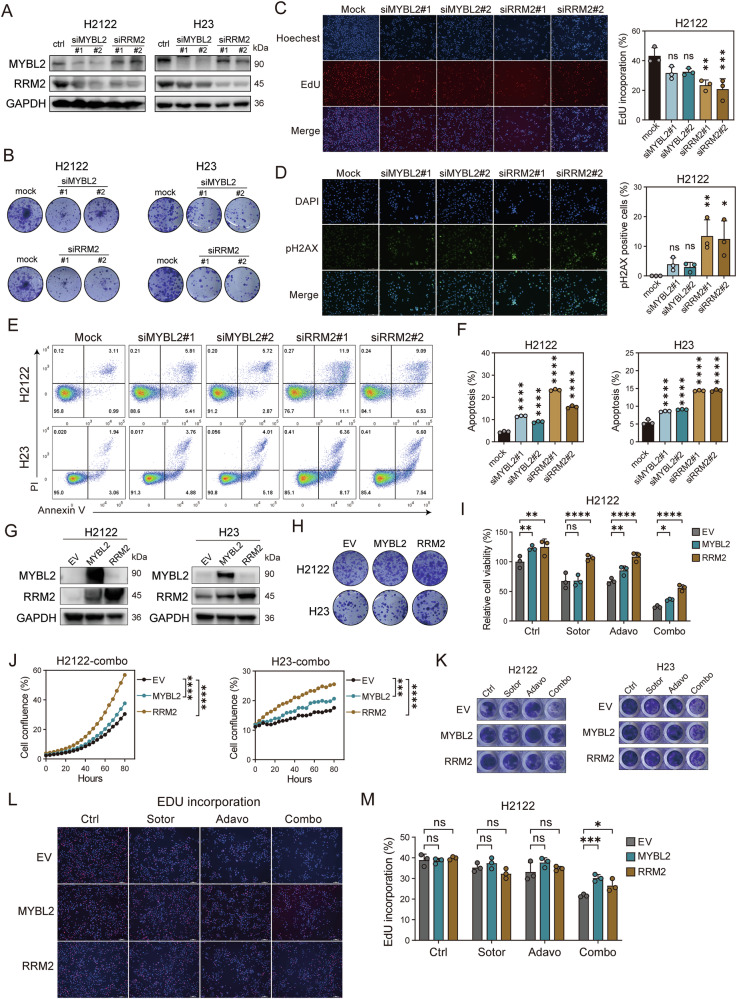
Overexpression of MYBL2 or RRM2 partially counteracts the synergistic inhibition. **A**The expression of MYBL2 and RRM2 proteins in H2122 and H23 cells under mock, siMYBL2 or siRRM2 treatment for 72 h. **B** Representative images of colony formation in H2122 and H23 cells under mock, siMYBL2 or siRRM2 treatment for 10–14 days. **C** Representative images and proportion of EdU incorporation assay in H2122 cells under mock, siMYBL2 or siRRM2 treatment for 48 h.*n* = 3 per group. **D** Representative images and proportion of γH2AX positive cells in H2122 cells under mock, siMYBL2 or siRRM2 treatment for 48 h. *n* = 3 per group. **E** Representative images of cell apoptosis assay in H2122 and H23 cells under mock, siMYBL2 or siRRM2 treatment for 48 h. **F** Cell apoptosis proportion in H2122 and H23 cells under mock, siMYBL2 or siRRM2 treatment for 48 h. *n* = 3 per group. **G** The expression of MYBL2 and RRM2 proteins in H2122 and H23 cells under empty vector (EV), MYBL2 or RRM2 plasmids treatment for 48 h. **H** Representative images of colony formation H2122 and H23 cells under EV, MYBL2 or RRM2 plasmids treatment for 10–14 days. **I** The cell viability of H2122 cells overexpressing EV, MYBL2, and RRM2 under control, sotorasib, adavosertib or combination treatment for 72 h. *n* = 3 per group. **J** The cell growth of H2122 and H23 cells overexpressing EV, MYBL2, and RRM2 under combination of sotorasib and adavosertib treatment for 72 h. **K** Representative images of colony formation in H2122 and H23 cells overexpressing EV, MYBL2, and RRM2 under combination of sotorasib and adavosertib treatment for 10-14 days. **L, M** Representative images (**L**) and proportion (**M**) of EdU positive cells in H2122 cells overexpressing EV, MYBL2, and RRM2 under control, sotorasib, adavosertib or combination treatment for 48 h. *n* = 3 per group. Data are mean ± SD of three independent replicates.

To investigate whether the inhibition of the MYBL2-RRM2 axis would replicate the biological effects observed with the combined treatment of sotorasib and adavosertib in our previous experiments, we utilized siRNA to knock down MYBL2 or RRM2 (Supplementary Fig. S6B). In H2122 and H23 cell lines, we found that knocking down MYBL2 reduced the expression of RRM2, whereas knocking down RRM2 did not affect the expression of MYBL2. This suggests that MYBL2 is upstream of RRM2, consistent with our previous results (Fig. 5A and Supplementary Fig. S6C). Additionally, we evaluated the effects of knocking down MYBL2 or RRM2 on cell viability, cell growth, long-term colony formation ability, cell proliferation, DNA damage, and cell apoptosis. By assessing cell viability, growth and long-term colony formation ability, we found that knocking down MYBL2 or RRM2 inhibited cell viability and growth (Fig. 5B and Supplementary Fig. S6D, E). Then, we examined the proportion of EdU incorporation and the proportion of γH2AX-positive cells, and the results indicated that siRNAs could reduce cell proliferation and increase DNA damage (Fig. 5C, D). Further analysis of cell apoptosis revealed that knocking down the expression of MYBL2 or RRM2 also increased apoptosis in H2122 and H23 cells (Fig. 5E, F). Notably, we observed that the inhibitory effect of knocking down MYBL2 on cellular biological functions was lower than that of RRM2, which is consistent with the expression levels of RRM2 we detected. We speculate that RRM2 is the key protein exerting the effect, and the degree of inhibition of RRM2 expression by sotorasib and adavosertib represents the strength of the synergistic inhibition effect of the two drugs.

Next, we performed gene overexpression of MYBL2 or RRM2 to observe the biological effects of the MYBL2-RRM2 axis. We used overexpression plasmids to overexpress MYBL2 or RRM2 in 293 T, H2122, and H23 cells. Overexpression of MYBL2 increased RRM2 expression, while overexpressing RRM2 had no effect on the expression of MYBL2, further confirming that MYBL2 is an upstream transcription factor of RRM2 (Fig. 5G and Supplementary Fig. S6F). We investigated the biological effects of overexpression in H2122 cells and observed that transfection with overexpression plasmids slightly enhanced cell viability and proliferation, without significantly impacting DNA damage or apoptosis. (Fig. 5H and Supplementary Fig. S6G–J).

Then, we aimed to determine whether the overexpression of the MYBL2-RRM2 axis could reverse the synergistic inhibitory effects of the combined treatment with WEE1i and G12Ci. To achieve this, we overexpressed MYBL2 or RRM2 under conditions of monotherapy or combination drug treatment of sotorasib and adavosertib in H2122 and H23 cells. The results of the cell viability assay indicated that overexpression of MYBL2 or RRM2 could partially reverse the inhibitory effects of the combination treatment, with the compensatory effect of RRM2 overexpression being stronger (Fig. 5I and Supplementary Fig. S7A). Additionally, cell growth experiments demonstrated that overexpression of MYBL2 or RRM2 could partially mitigate the inhibitory effects of the combination treatment (Fig. 5J). The results of long-term colony formation ability further confirmed this conclusion (Fig. 5K and Supplementary Fig. S7B). We then assessed the proportion of EdU incorporation, and the results revealed that overexpression of MYBL2 or RRM2 increased the proportion of EdU-incorporating cells in the combination treatment group, indicating a partial reversal of the inhibitory effect (Fig. 5L, M). Analysis of apoptosis supported these findings, showing that overexpression of the MYBL2-RRM2 axis reduced the proportion of apoptotic cells in the combination treatment group in H2122 and H23 cells (Supplementary Fig. S7C, D).

In summary, the above results demonstrate that the biological effects elicited by the inhibition of the MYBL2-RRM2 axis are consistent with those observed in previous experiments with the combined drugs, and the overexpression of the MYBL2-RRM2 axis can partially reverse the inhibitory effects of the combination treatment, providing compelling evidence that the MYBL2-RRM2 axis is a critical pathway for exerting synergistic inhibitory effects.

### Replenishment of dNTPs reverses the synergistic inhibitory effect

**Fig. 6:**
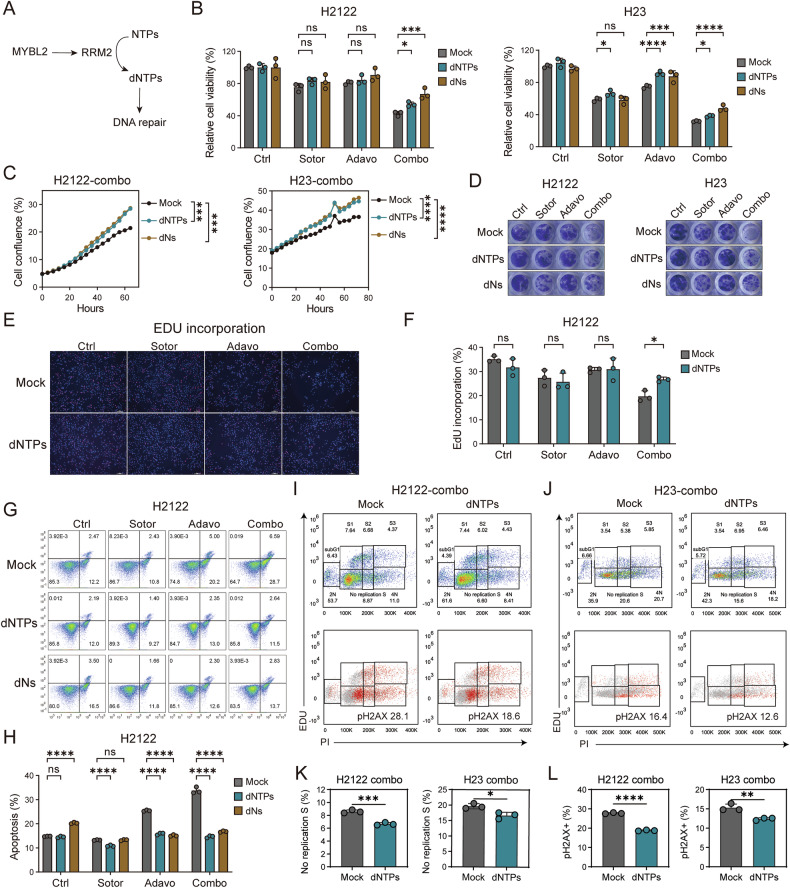
Replenishment of dNTPs reverses the synergistic inhibitory effect. **A** Diagram illustrating the function of RRM2. **B** The cell viability of H2122 and H23 cells under control, sotorasib, adavosertib or combination treatment for 72 h with and without 10 μM dNTPs or dNs. *n* = 3 per group. **C** The cell growth of H2122 and H23 cells under combination of sotorasib and adavosertib treatment for 72 h with and without 10 μM dNTPs or dNs. **D** Representative images of colony formation in H2122 and H23 cells under combination of sotorasib and adavosertib treatment for 10–14 days with and without dNTPs or dNs. **E, F** Representative images (**E**) and proportion (**F**) of EdU positive in H2122 cells under control, sotorasib, adavosertib or combination treatment for 48 h with and without 10 μM dNTPs. *n* = 3 per group. **G, H** Representative images (**G**) and apoptosis proportion (**H**) of cell apoptosis assay in H2122 cells under control, sotorasib, adavosertib or combination treatment for 48 h with and without 10 μM dNTPs or dNs. *n* = 3 per group. **I, J** Representative images of cell cycle distribution and γH2AX positive cells in H2122 (**I**) and H23 (**J**) cells under combination treatment for 48 h with and without 10 μM dNTPs performed using flow cytometry. The cell cycle was divided into the 2 N, 4 N, S1, S2, S3, no replication S, and subG1 phases by PI and EdU. **K** Proportion of no replication S phase in H2122 and H23 cells under combination treatment for 48 h with and without dNTPs. *n* = 3 per group. **L** Proportion of γH2AX positive cells in H2122 and H23 cells under combination treatment for 48 h with and without dNTPs. *n* = 3 per group. Data are mean ± SD of three independent replicates.

RRM2 is an enzyme responsible for converting NTPs into dNTPs within cells, thereby maintaining the dNTPs content and aiding in DNA repair [42]. Inhibition of RRM2 expression leads to the depletion of the dNTPs pool, which in turn contributes to DNA replication stress and DNA damage [42, 43] (Fig. 6A). Given that we previously identified RRM2 as a key protein in exerting synergistic effects, we aimed to determine whether replenishing RRM2 enzymatic product, dNTPs, could reverse this inhibitory effect. In some literature, due to the polarity of dNTPs, dNs have been used as the product of RRM2 to accomplish experiments [37, 38]. Therefore, we utilized both dNTPs and dNs to verify our hypothesis.

We added 10 μM of dNTPs or dNs to the single or combination treatment groups of sotorasib and adavosertib in H2122 and H23 cells. The addition of dNs or dNTPs did not affect cell viability on their own, but increased cell viability in the combination treatment group, demonstrating their ability to reverse the inhibition of cell viability caused by the combined drugs (Fig. 6B). This was also reflected in cell growth (Fig. 6C). The results of long-term colony formation indicated that both dNTPs and dNs can reverse the inhibition of colony formation ability caused by the combined drugs without affecting the intrinsic colony formation ability of the cells, demonstrating that dNTPs are key to the synergistic effects of WEE1i and G12Ci (Fig. 6D and Supplementary Fig. S8A). Consistent with the results of cell viability and long-term growth, we found that the impact of dNTPs on cell proliferation was primarily observed in the combination treatment group, with no significant effect on untreated cells (Fig. 6E, F). Moreover, the assessment of apoptosis levels indicated that dNs or dNTPs can significantly reduce the apoptosis levels in H2122 and H23 cells under the synergistic inhibition of the two drugs (Fig. 6G, H and Supplementary Fig. S8B, C). These results collectively demonstrate that dNTPs (or dNs) are key molecules for the synergistic inhibitory effect of the combined drugs.

Flow cytometry was used to analyze the proportions and distribution of EdU, PI, and γH2AX in H2122 and H23 cells after 24 h of treatment with or without dNTPs (Fig. 6I, J and Supplementary Fig. S8D, E). In our cell cycle analysis, we observed that the inclusion of dNTPs significantly reduced the proportion of no replication S phase cells within the combination treatment group. This result indicates that dNTPs play a crucial role in alleviating DNA replication stress (Fig. 6K and Supplementary Fig. S8F). Furthermore, our detection of γH2AX proportions revealed that dNTPs primarily alleviate DNA damage in the combination treatment group. This finding confirms that dNTPs can reverse the synergistic effect of WEE1i and G12Ci on DNA damage, which is triggered by DNA replication stress (Fig. 6L and Supplementary Fig. S8G). These observations highlight the potential of dNTPs in mitigating the adverse effects of combined drug treatments on cellular DNA replication.

In summary, the addition of dNTPs not only reduces the proportion of no replication S phase cells, thereby alleviating DNA replication stress, but also significantly decreases the level of DNA damage in combination drug treatments. These results underscore the importance of dNTPs as key molecular agents in counteracting the synergistic inhibitory effects of WEE1i and G12Ci, providing valuable insights into their molecular theoretical basis.

### Wee1 and G12C co-inhibition significantly suppresses lung cancer growth in vivo

**Fig. 7:**
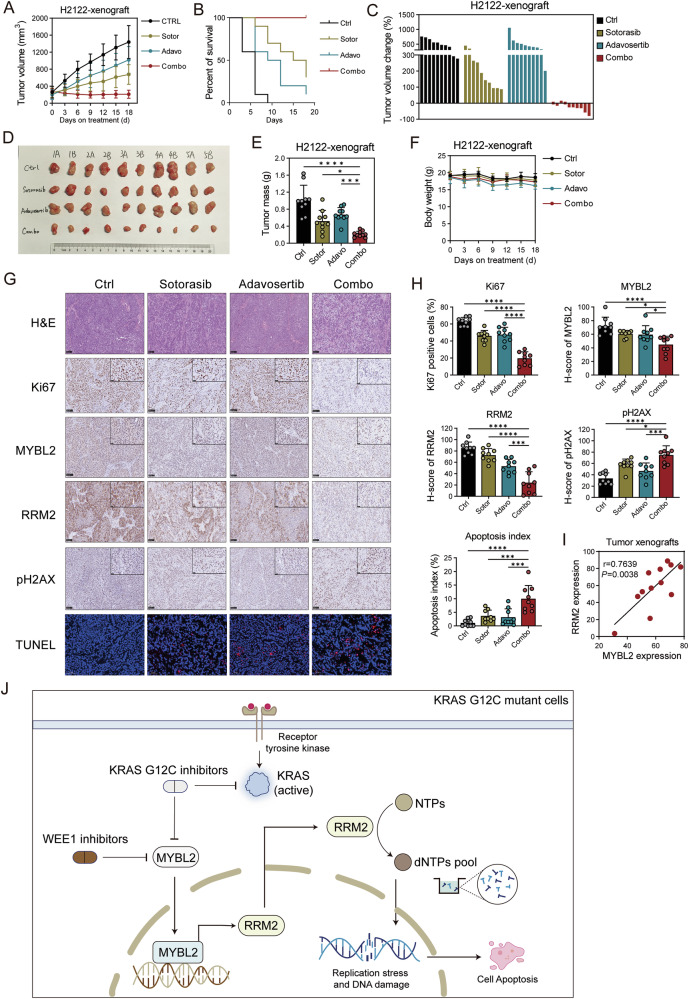
Wee1 and G12C c0-inhibition significantly suppresses lung cancer growth in vivo*.* **A** H2122 xenografts tumors treated with sotorasib, adavosertib, or a combination of both for 18 days shown in tumor growth. *n* = 5 mice, with 10 tumors per group. **B** Percent survival in H2122 xenografts tumors treated with sotorasib, adavosertib, or their combination for 18 days. Tumor volume exceeding 600 mm^3^ is considered lethal. *n* = 10 tumors per group. **C** Waterfall plot analyses of changes in tumor volume at the end of treatment. *n* = 10 tumors per group. **D** Photograph of xenografts tumors treated with sotorasib, adavosertib, or a combination of both for 18 days. *n* = 10 tumors per group. **E** Tumor mass of xenografts tumors treated with sotorasib, adavosertib, or a combination of both for 18 days. *n* = 10 tumors per group. **F** Body weight of mice bearing H2122 xenografts tumors treated with sotorasib, adavosertib, or a combination of both for 18 days. *n* = 5 mice per group. **G** H&E staining, IHC staining, and TUNEL staining of xenografts tumors. Scale bar, 100 μm in the full image and 25 μm in the magnified image of the top right corner. For TUNEL staining, The blue fluorescence (DAPI) in tumor represented for tumor cells and red fluorescence (TUNEL, TMR red) represented for apoptosis tumor cells. Scale bar, 50 μm. **H** The staining expression of Ki67, MYBL2, RRM2, pH2AX and apoptosis index in xenografts tumors treated with sotorasib, adavosertib, or their combination for 18 days. *n* = 9 per group. Expression is calculated using three slices per group, with three randomly selected regions per slice. **I** The correlation between MYBL2 and RRM2 IHC H score in xenografts tumors. *n* = 12. **J** Abstract. In KRAS-G12C mutant NSCLC, the combination of G12Ci and WEE1i synergistically suppresses the MYBL2-RRM2 axis. This suppression leads to a reduction in dNTPs, triggers DNA replication stress, and results in the accumulation of DNA damage, ultimately culminating in apoptosis. Data are mean ± SD of independent replicates.

We evaluated the in vivo therapeutic efficacy of KRAS G12C inhibitor sotorasib and WEE1 inhibitor adavosertib in mouse xenograft models. Mice bearing H2122 xenografts were treated with sotorasib, adavosertib, or a combination of both for 18 days, monitoring tumor growth and mouse weight (Fig. 7A, B). Interestingly, adavosertib monotherapy did not significantly suppress tumor growth, possibly due to the study’s concentration of 40 mg/kg, which is lower than 60 mg/kg used in previous literature for monotherapy in mouse models [44–46], thus lacking strong tumor-inhibiting effects. However, notably, the combination of low concentration adavosertib with sotorasib significantly inhibited tumor growth, matching the synergistic effects observed in vitro (Fig. 7A, B). Waterfall plot analysis of changes in tumor volume at the end of the treatment showed that tumors in the combination treatment group regressed by approximately 20%, whereas monotherapy groups did not achieve tumor inhibition (Fig. 7C). The combination treatment of adavosertib and sotorasib significantly inhibited tumor size and mass but did not cause mouse body weight loss (Fig. 7D–F). These findings indicate that the combination therapy of sotorasib and adavosertib effectively inhibits tumor growth through a synergistic effect.

We performed IHC staining on xenograft tumors to assess the expression of Ki-67, MYBL2, RRM2, and γH2AX, and conducted TUNEL staining to evaluate apoptosis levels. The combination treatment of adavosertib and sotorasib exhibited pronounced effects in IHC analyses. Notably, adavosertib enhanced the anti-proliferative effect of sotorasib on tumor cells, as indicated by reduced Ki-67 staining (Fig. 7G, H). The combination group showed the lowest H-scores for MYBL2 and RRM2, and the highest H-score for γH2AX. These findings suggest that the in vivo combination of sotorasib and adavosertib significantly suppresses the MYBL2-RRM2 axis while increasing γH2AX expression (Fig. 7G, H). Consistent with the involvement of this axis, combination treatment also markedly elevated apoptosis levels in tumor tissues (Fig. 7G, H). Furthermore, we validated the correlation between MYBL2 and RRM2 in the xenograft model. IHC analysis of subcutaneous xenografts revealed a positive correlation between MYBL2 and RRM2 expression, in agreement with our in vitro findings and bioinformatic analyses (Fig. 7I).

Figure 7J summarizes the overall findings, demonstrating that in KRAS-G12C mutant NSCLC, the combination of G12Ci sotorasib and WEE1i adavosertib synergistically suppresses the MYBL2-RRM2 axis. This suppression leads to a reduction in dNTPs, triggers DNA replication stress, and results in the accumulation of DNA damage, ultimately culminating in apoptosis. In conclusion, our findings suggest that the combination of sotorasib and adavosertib offers a more effective therapeutic strategy for KRAS-G12C mutated NSCLC.

## Discussion

In the CodeBreak 200 and KRYSTAL-12 phase III trial, the mPFS in patients with previously treated advanced/metastatic NSCLC harboring a KRAS G12C mutation with sotorasib and adagrasib were 5.6 months and 5.49 months, respectively, indicating limited efficacy as monotherapies [15, 16]. In addition to the increasing number of KRAS G12C inhibitors, G12D inhibitors [47, 48] and pan-KRAS inhibitors [49, 50] are also under development or entering clinical trials. In the disclosed monotherapy trials, these drugs have not shown a surprising improvement in efficacy. Therefore, there is an urgent need for a good combination treatment strategy for KRAS mutations that can improve efficacy without significantly increasing toxic side effects. Our findings demonstrate that WEE1 inhibitors synergize with the KRAS G12C inhibitor sotorasib, providing a potential clinical application strategy for KRAS-targeted drugs. Extending this approach to other KRAS G12C inhibitors and even pan-KRAS inhibitors holds substantial potential for clinical applications.

WEE1 inhibitors act as chemo-sensitizers, exhibiting synergistic activity with DNA-damaging agents, including various chemotherapeutics and radiotherapy, in preclinical models. Adavosertib, a WEE1 inhibitor, induces S and G2/M cell cycle checkpoint override, leading to DNA damage accumulation and replicative or mitotic catastrophe. This mechanism enhances the cytotoxicity of chemo/radiotherapy by promoting cell cycle disruption, impairing DNA repair, and inducing apoptosis [24]. Additionally, WEE1 inhibitors also demonstrate synergistic effects with various targeted therapies in KRAS-mutated cancers [26–31]. In our research, WEE1 inhibitors can also exert synergistic effects with KRAS G12C inhibitors, enhancing the efficacy of the targeted therapy. The synergistic effects are achieved by undergoing DNA replication stress, and consequent accumulation of DNA damage, ultimately leading to cell cycle perturbation and apoptosis. These findings highlight their potential in combination strategies to improve therapeutic efficacy.

Notably, the clinical development of adavosertib has been hindered by dose-limiting toxicities, particularly hematological (e.g., neutropenia, anemia), as reported in multiple clinical trials (NCT04590248, NCT02151292, NCT03284385) [32–34]. Consequently, we focused on evaluating the potential for adavosertib dose reduction. We assessed drug sensitivity across multiple normal cell types, particularly human monocyte-macrophage (THP-1) and T-cell (Jurkat) lines. For in vitro experiments, adavosertib concentrations of 250 nM (H2122 cells) and 200 nM (H23 cells) were employed - concentrations that demonstrate minimal toxicity in most normal cell types. In vivo, we administered adavosertib at 40 mg/kg, which is lower than the commonly used preclinical doses [44–46]. Despite the reduced dose, we observed significant synergistic anti-tumor activity and no notable toxicity, as evidenced by stable body weight and vitality in treated mice. These findings also provide clinical safety data supporting the strategy of using low-dose WEE1 kinase inhibitors as sensitizers for KRAS G12C inhibitors in clinical settings.

Our research identifies the MYBL2–RRM2–dNTPs pathway as central to the synergistic effect of WEE1 and KRAS G12C inhibitors in KRAS G12C-mutant NSCLC. RRM2 participates in nucleotide metabolism and maintains the dNTPs pools necessary for DNA biosynthesis, repair, and replication [39]. As a product of RRM2 enzymatic activity, dNTPs pools play a role in replication stress, the DNA damage response, and execution of senescence pathways after oncogene activation [42]. The decrease in dNTPs pools leads to replication stress, accumulation of DNA damage and a sustained induction of the DDR [43]. Regarding the upstream regulatory elements of RRM2, our research indicates that MYBL2 is the most relevant transcription factor that regulates the transcription of RRM2, consistent with the conclusions of other studies [41]. Multiple studies have shown that the mechanism by which WEE1 kinase, as a combination therapy drug, works is by reducing the level of RRM2, leading to dNTPs depletion, reducing DNA repair, and causing replication stress, thereby increasing apoptosis [41, 51–57]. In our study, RRM2 is also the key protein for the synergistic inhibitory effect of WEE1 kinase and G12C inhibitors. Combination therapy significantly disrupts the MYBL2–RRM2 axis, with overexpression of MYBL2 or RRM2, or dNTPs replenishment, partially rescuing the inhibitory effect. This mechanism provides a theoretical basis for combining WEE1 and KRAS G12C inhibitors in targeted lung cancer therapy.

Developing combination therapy strategies for KRAS-mutant lung cancer is crucial to achieving more durable clinical outcomes. The current combination strategies, including the integration with chemotherapy, immunotherapy, and targeted therapy, fail to enhance efficacy without increasing toxicity [20–22]. Due to the strong synergistic effects observed between WEE1 inhibitors and G12C inhibitors in in vitro cell models, we further validated the efficacy and safety of the combination strategy involving WEE1i and G12Ci in xenograft tumor models in vivo. Low concentrations of the WEE1 inhibitor were used to investigate its sensitizing effect on the G12C inhibitor. Our results demonstrated that WEE1 inhibitor at lower concentrations did not significantly inhibit tumor growth on its own, but significantly enhanced the inhibitory effects of the G12C inhibitor. The combined use of both drugs markedly inhibited tumor growth without causing significant toxicity. Overall, combining these two drugs for treating KRAS G12C-mutant lung cancer is feasible and promising.

Our study has several limitations. While we examined the impact of combination therapy on cell cycle-related proteins via western blot, we did not provide a detailed explanation and clarification on whether certain cell cycle-related proteins might also be involved in this synergistic effect. Previous studies had suggested CDK1/2 inhibition could lead to RRM2 degradation pathway [58], and we observed the lowest p-CDK1 levels in combination therapy, which may partially drive RRM2 degradation. However, transcriptional data showed a significant synergistic reduction in RRM2, so the role of degradation was not fully explored. Sequencing data suggested that E2F targets and the G2M checkpoint as key pathways, highlighting MYBL2 and RRM2 as critical factors, yet overexpression and dNTPs replenishment only partially restored the effect. Additionally, the continuous emergence of new KRAS G12C inhibitors, even KRAS G12D and pan-KRAS inhibitors provides new treatment options not tested in our study. Nonetheless, our findings with WEE1 inhibitors and Sotorasib suggest WEE1 inhibitors may enhance the efficacy of KRAS-targeted therapies, improving clinical outcomes in KRAS-mutant lung cancer.

In summary, we confirmed a strong synergistic effect between WEE1 inhibitors (WEE1i) and KRAS G12C inhibitors (G12Ci) in KRAS G12C-mutant lung cancer, driven primarily by the MYBL2–RRM2 axis. This combination induces extensive DNA replication errors, cell cycle arrest, and DNA damage, ultimately leading to apoptosis. Overexpression of the MYBL2–RRM2 axis or dNTPs replenishment partially reverses this effect, suggesting an important contributory role of this axis. Our findings suggest that the dual inhibition strategy may offer a promising and tolerable combinatorial strategy for KRAS G12C-mutant lung cancer.

## Supplementary information

Supplementary materials

WB raw data

## Data Availability

RNA-seq data has been deposited to CNCB-NGDC database (HRA012323). Data from this study are available upon reasonable request to the corresponding author.
