# Supplementary material for: WEE1 inhibitor exerts synergistic effect with KRAS G12C inhibitor via MYBL2-RRM2 axis in KRASG12C-mutant lung cancer
Source: Cell Death Dis. 2025 Aug 30;16(1):661. doi: 10.1038/s41419-025-07992-4 (PMC12398517; doi:10.1038/s41419-025-07992-4)
Supplement: Supplementary file 1 — Supplementary materials [file 41419_2025_7992_MOESM1_ESM.pdf]

Supplementary Materials for

WEE1 inhibitor exerts synergistic effect with KRAS G12C inhibitor via MYBL2-RRM2 axis in $KRAS^{G12C}$-mutant lung cancer

This PDF file includes:

Supplementary Figures and Legends (Figures. S1 to S8)
Supplementary Tables S1 to S5

Supplementary Figures and Legends

Figure. S1

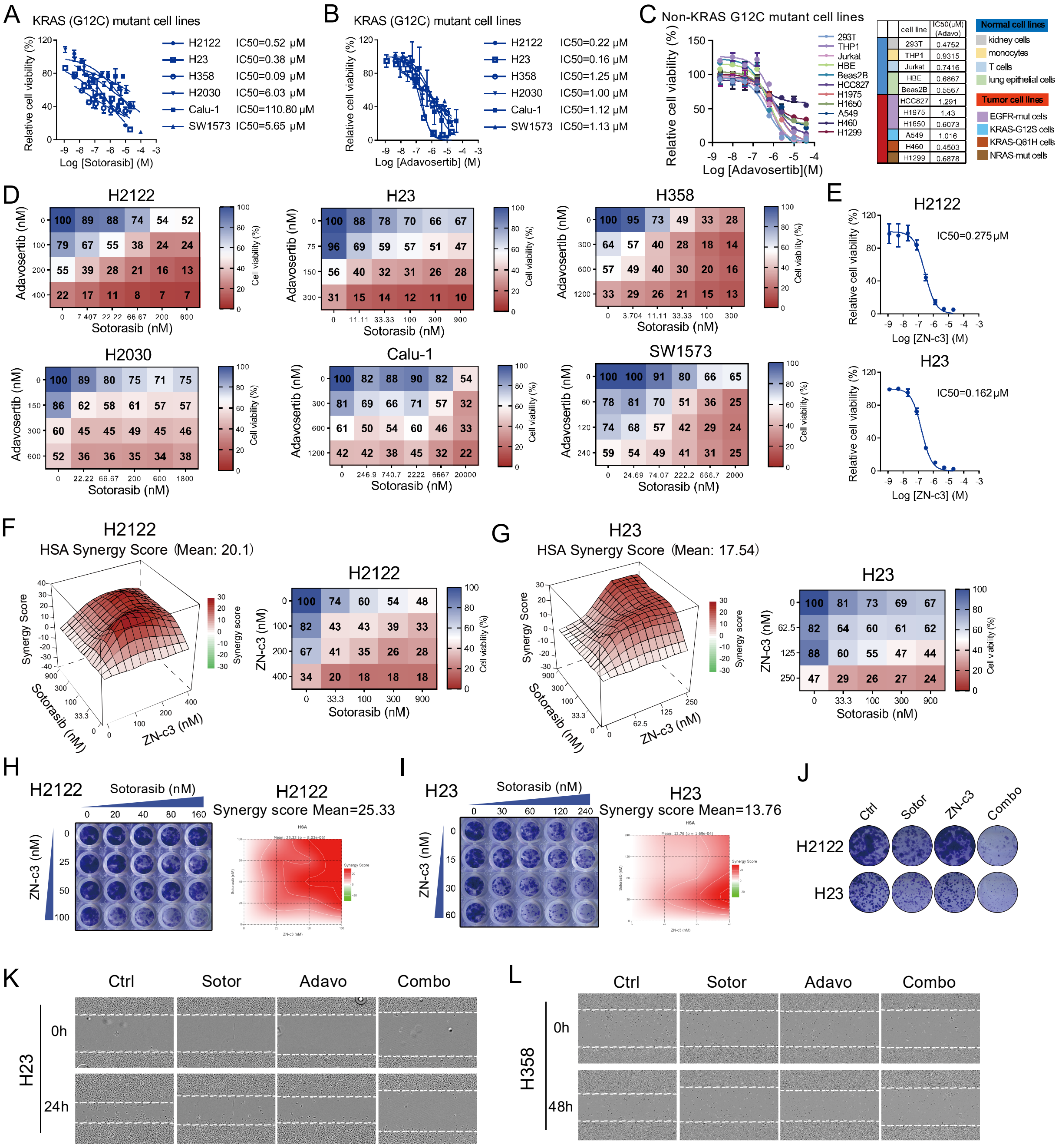

Figure. S1 WEE1 inhibitor exhibits synergistic effects with KRAS G12C inhibitor

A and B. Dose-response curves and IC50 of KRAS G12C mutant cell lines treated with sotorasib (A) and adavosertib (B) for 72 hours. C. Dose-response curves and IC50 of non-KRAS G12C mutant cell lines treated with adavosertib. D. The relative cell viability with gradient concentrations of sotorasib and adavosertib in H2122, H23, H358, H2030, Calu-1, and SW1573

cells for 72 hours. E. Dose-response curves and IC50 of H2122 and H23 cells treated with ZN-c3 for 72 hours. F and G. HSA synergy maps for the combination of sotorasib and ZN-c3 in H2122 (F), H23 (G) cells. H and I. Colony formation assay and HSA synergy score of H2122 (H) and H23 (I) cell lines treated with gradient concentrations of sotorasib and ZN-c3 for 10-14 days. J. Representative images of colony formation in H2122, H23 cells under untreated, sotorasib, ZN-c3 or combination treatment conditions. K and L. Representative images of cell scratch assay in H23 (K) and H358 (L) under treatment. Data are mean ± SD of three independent replicates.

Figure. S2

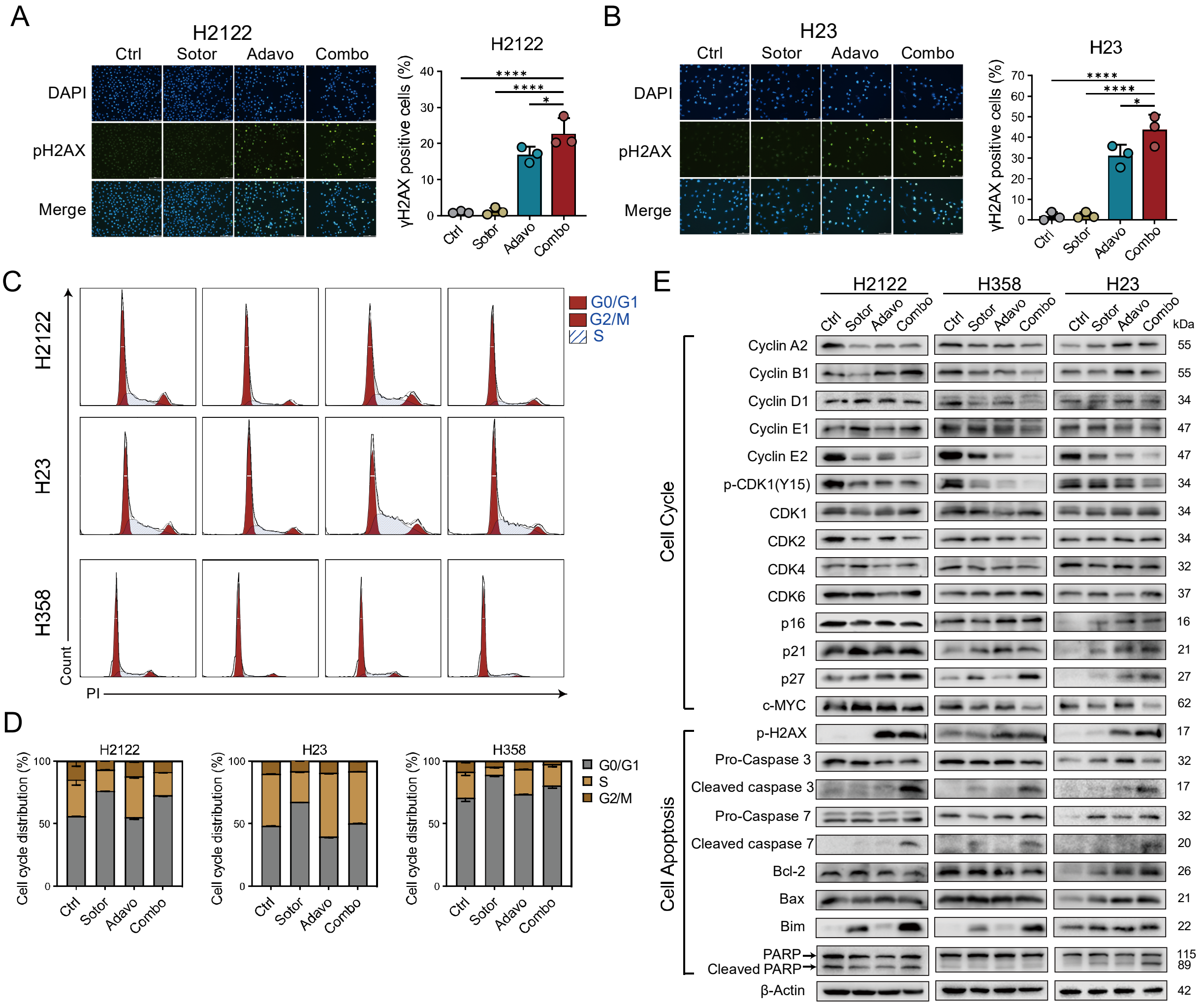

Figure. S2 Combination of G12Ci and WEE1i induces DNA damage and changes in apoptosis-related proteins and cell cycle-related proteins

A and B. Representative images and proportion of γH2AX positive cells in H2122 (A) and H23 (B) cells under treatment using γ-H2AX Immunofluorescence. n = 3 per group. C. Representative images of cell cycle assay in H2122, H23 and H358 cells under treatment for 24 hours. The cell cycle was divided into the G0/G1, S and G2/M phases. D. Cell cycle distribution in H2122, H23 and H358 cells under treatment for 24 hours. n = 3 per group. E. The expression of proteins related to the cell cycle and apoptosis in H2122, H23 and H358 cells under treatment for 24 hours by western blot analysis.

Figure. S3

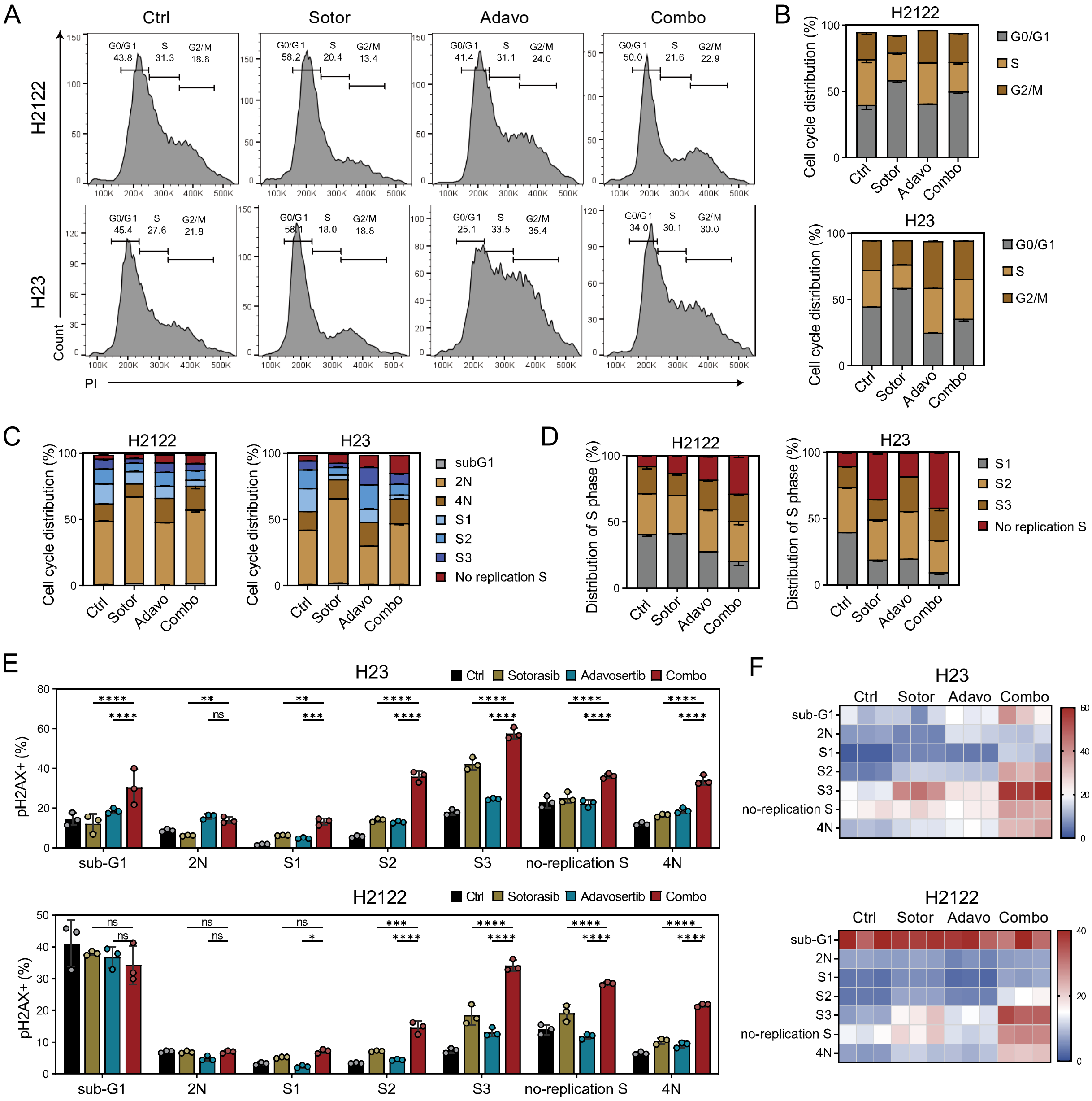

Figure. S3 Combination of G12Ci and WEE1i induces cell cycle perturbation, replication stress, and DNA damage, leading to apoptosis

A. Representative images of cell cycle assay in H2122, H23 and H358 cells under treatment for 48 hours by using PI and EdU. The cell cycle was divided into the G0/G1, S and G2/M phases. B. Cell cycle distribution in H2122, H23 and H358 cells under treatment for 48 hours by using PI and EdU. n = 3 per group. C. Proportion of cell cycle distribution in H2122 and H23 cells under treatment. The cell cycle was divided into the 2N, 4N, S1, S2, S3, no replication S, and subG1 phases by PI and EdU. n = 3 per group. D. Proportion of no replication S phase in S phase in H2122 and H23 cells under treatment for 48 hours. n = 3 per group. E. The proportion of γH2AX positive cells in each phase of H2122 and H23 cells under treatment using flow

cytometry. n = 3 per group. F. Heatmap of the proportion of γH2AX positive cells in each cell cycle phase. Data are mean ± SD of three independent replicates.

Figure. S4

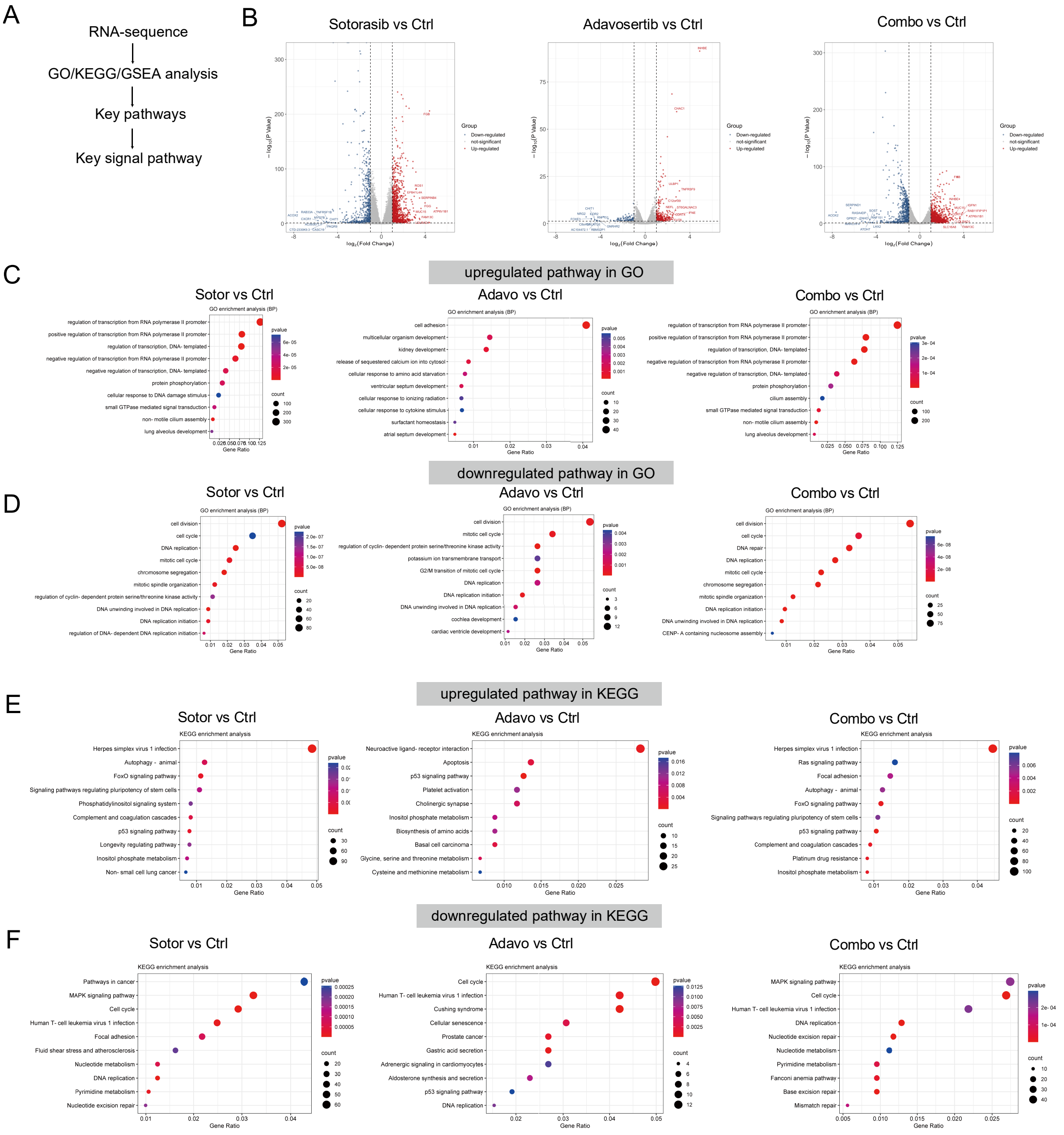

Figure. S4 Volcano plot, GO, and KEGG analysis performed using RNA-seq data

A. RNA-seq data processing workflow. B. Volcano plot of differentially expressed genes (DEGs) comparing sotorasib, adavosertib and combination groups to the control group derived from RNA-seq data. C and D. GO analysis of up-regulated (C) and down-regulated (D) DEGs comparing sotorasib, adavosertib and combination groups to the control group derived from RNA-seq data. E and F. KEGG analysis of up-regulated (E) and down-regulated (F) DEGs comparing sotorasib, adavosertib and combination groups to the control group derived from RNA-seq data.

Figure. S5

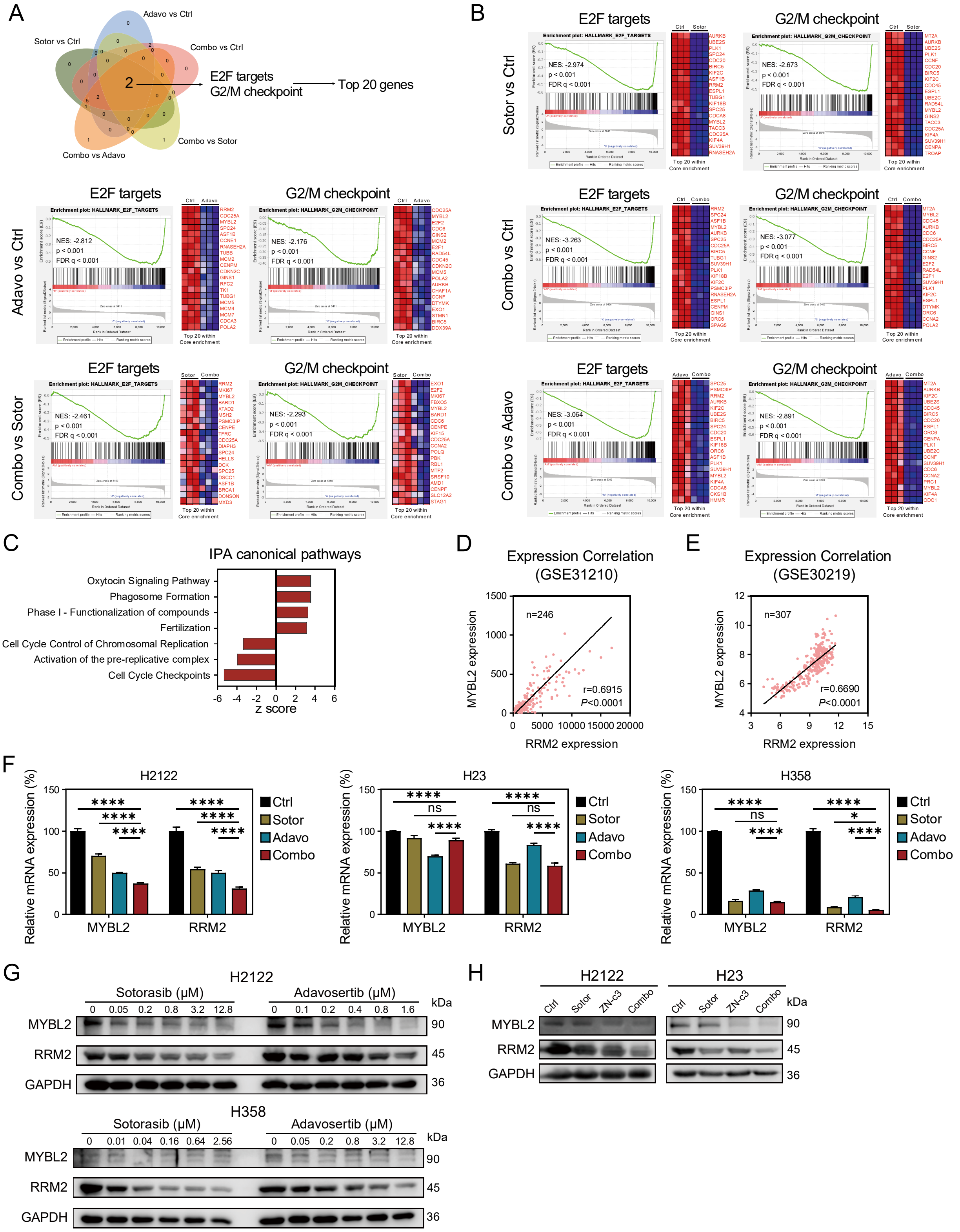

Figure. S5 The synergistic effect of WEE1i and G12Ci may depend on MYBL2-RRM2 pathway

A. Venn diagram of down-regulated pathways from GSEA analysis comparing sotorasib, adavosertib, and combination groups to the control group, as well as comparing the combination group to sotorasib and adavosertib groups. B. NES images, values and top 20 genes of E2F targets and G2M checkpoint pathways in GSEA analysis, with pairwise comparisons among the four treatment groups. C. IPA canonical pathway analysis comparing combination groups to control group. D and E. The correlation between MYBL2 and RRM2 in GSE31210 (D) and our GSE30219 (E). F. The expression of MYBL2 and RRM2 mRNA levels in H2122, H23 and H358 cells under control, sotorasib, adavosertib or combination treatment for 24 hours. G. The expression of MYBL2 and RRM2 proteins in H2122 and H358 cells treated with gradient concentrations of sotorasib and adavosertib for 24 hours. H. The expression of MYBL2 and RRM2 proteins in H2122 and H23 cells under control, sotorasib, ZN-c3 or combination treatment for 24 hours. Data are mean ± SD of three independent replicates.

Figure. S6

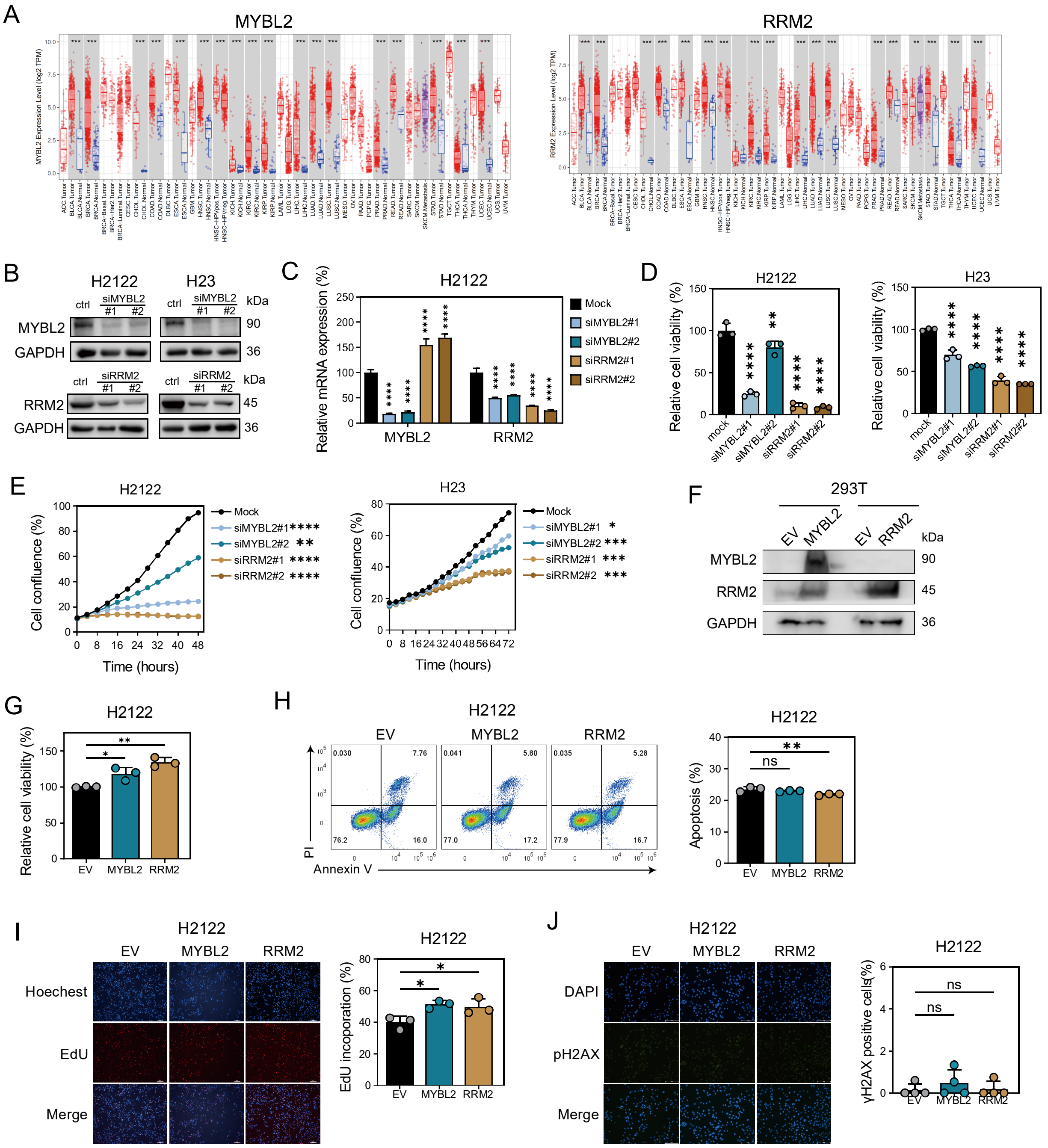

Figure. S6 Biological effects of targeting MYBL2-RRM2 axis in KRAS-G12C mutated cells

A. The expression of MYBL2 and RRM2 between cancerous tissue and adjacent non-cancerous tissue in TCGA datasets. B. The protein expression levels of MYBL2 or RRM2 genes in H2122 and H23 after 48 hours of knockdown using siRNAs. C. The mRNA expression of MYBL2 and RRM2 mRNA levels in H2122 cells under mock, siMYBL2 or siRRM2 treatment for 48 hours. D. The cell viability in H2122 and H23 cells under mock, siMYBL2 or siRRM2 treatment for 72 hours. E. The cell growth in H2122 and H23 cells under mock, siMYBL2 or siRRM2 treatment

for 72 hours. F. The expression of MYBL2 and RRM2 proteins in 293T cells under EV, MYBL2 or RRM2 plasmids treatment for 48 hours. G. The cell viability in H2122 cells under EV, MYBL2 or RRM2 plasmids treatment for 72 hours. H. Representative images and apoptosis proportion of cell apoptosis assay in H2122 cells under EV, MYBL2 or RRM2 plasmids treatment for 48 hours. I. Representative images and proportion of EdU positive cells of EdU incorporation assay in H2122 cells under EV, MYBL2 or RRM2 plasmids treatment for 48 hours. J. Representative images and proportion of γH2AX positive cells in H2122 cells under EV, MYBL2 or RRM2 plasmids treatment for 48 hours. Data are mean ± SD of three independent replicates.

Figure. S7

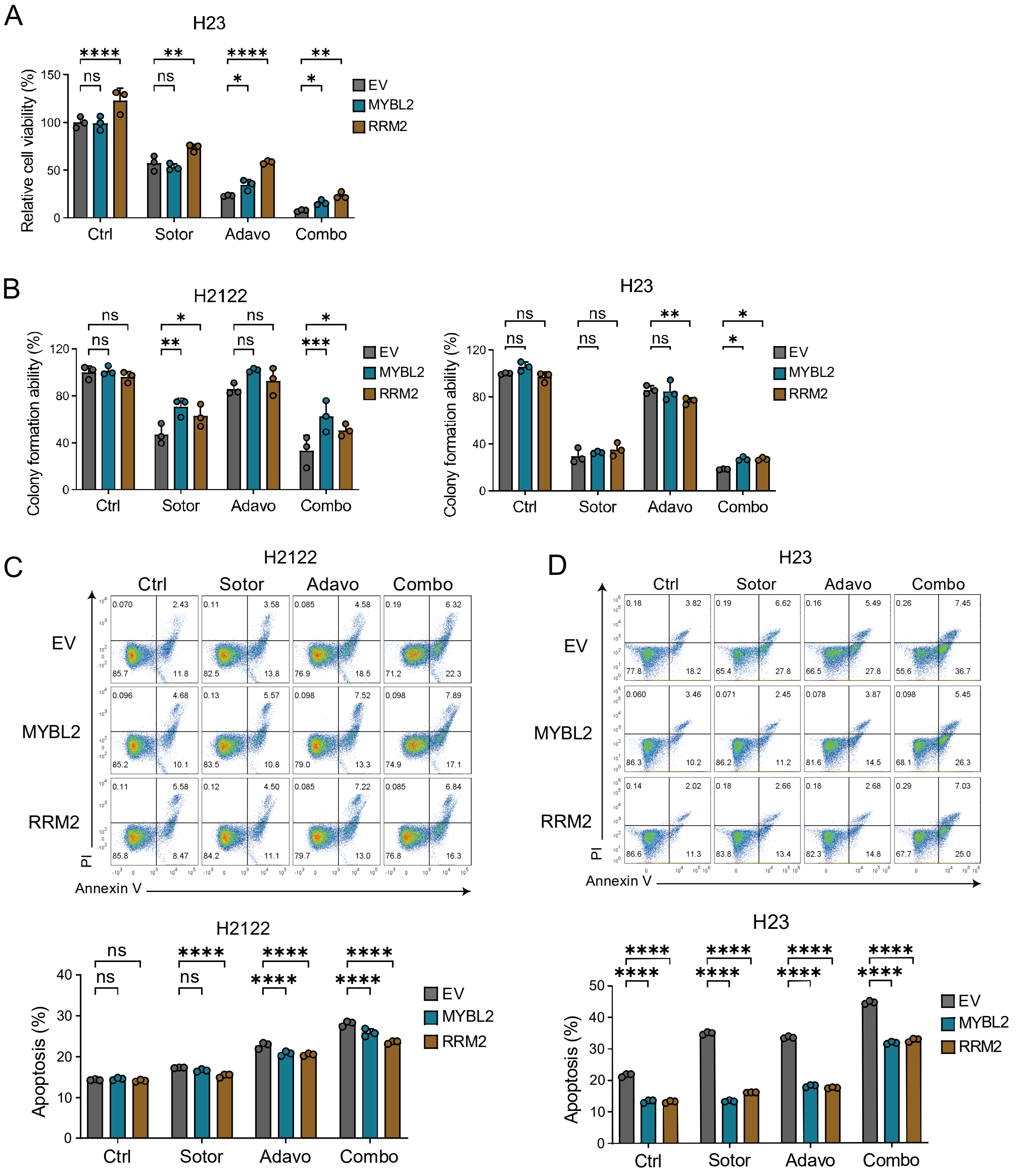

Figure. S7 Overexpression of MYBL2 or RRM2 can partially counteract the synergistic inhibition

A. The cell viability of H23 cells overexpressing EV, MYBL2, and RRM2 under control, sotorasib, adavosertib or combination treatment for 72 hours. n=3 per group. B. Colony formation ability in H2122 and H23 cells overexpressing EV, MYBL2, and RRM2 under

combination of sotorasib and adavosertib treatment for 10-14 days. n=3 per group. C and D. Representative images and apoptosis proportion of cell apoptosis assay in H2122 (C) and H23 (D) cells overexpressing EV, MYBL2, and RRM2 under control, sotorasib, adavosertib or combination treatment for 48 hours. n=3 per group. Data are mean ± SD of three independent replicates.

Figure. S8

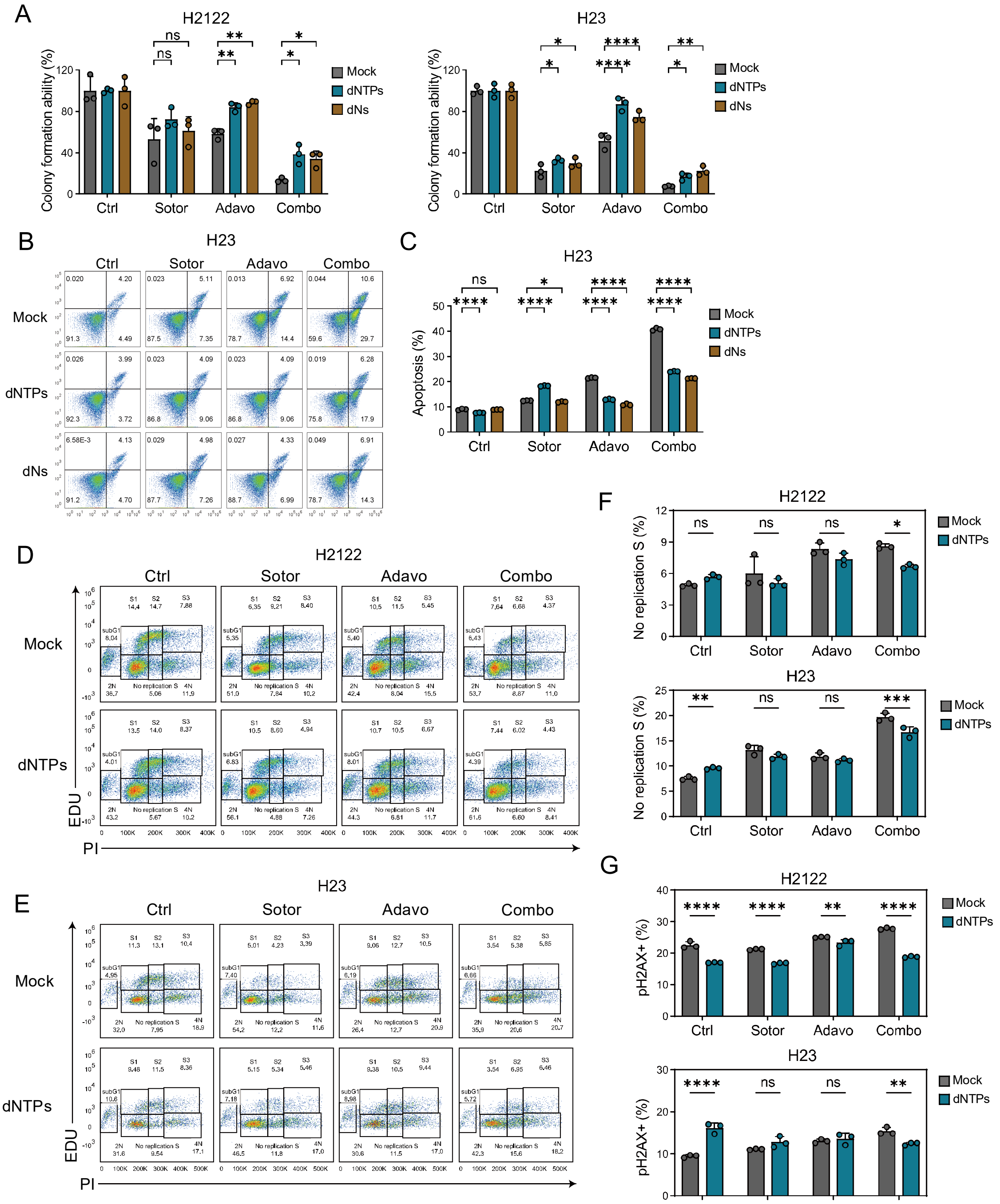

Figure. S8 Replenishment of dNTPs could also reverse the synergistic inhibitory effect

A. Colony formation ability in H2122 and H23 cells under combination of sotorasib and adavosertib treatment for 10-14 days with and without dNTPs or dNs. n=3 per group. B and C. Representative images (B) and apoptosis proportion (C) of cell apoptosis assay in H23 cells under control, sotorasib, adavosertib or combination treatment for 48 hours with and without dNTPs or dNs. n=3 per group. D and E. Representative images of cell cycle distribution in H2122 (D) and H23 (E) cells under treatment for 48 hours with and without dNTPs performed using flow cytometry. The cell cycle was divided into the 2N, 4N, S1, S2, S3, no replication S, and subG1 phases by PI and EdU. F. Proportion of no replication S phase in H2122 and H23 cells under control, sotorasib, adavosertib or combination treatment for 48 hours with and without dNTPs. n = 3 per group. G. Proportion of γH2AX positive cells in H2122 and H23 cells under control, sotorasib, adavosertib or combination treatment for 48 hours with and without dNTPs. n = 3 per group. Data are mean ± SD of three independent replicates.

Tables S1 to S5

Supplementary Table 1. Origin and key features of cell lines used in this study

**Cell line**	**Age**	**Gender**	**Disease**	***KRAS***	***TP53* status**	**Other**
H2122	46y	Female	Lung adenocarcinoma	p.Gly12Cys	p.Gln16Leu; p.Cys176Phe	
H23	51y	Male	Lung adenocarcinoma	p.Gly12Cys	p.Met246Ile	ATM: p.Gln1919Pro; STK11: p.Trp332Ter
H358	unspecified	Male	Lung adenocarcinoma	p.Gly12Cys	deletion	CTNNB1: p.Thr75Ala
H2030	unspecified	Male	Lung adenocarcinoma	p.Gly12Cys	p.Gly262Val	
Calu-1	47y	Male	Lung squamous cell carcinoma	p.Gly12Cys	deletion	TERT: c.1-124C>T
SW1573	44y	Female	Lung adenocarcinoma	p.Gly12Cys		CDKN2A: deletion; SMAD4: deletion; CTNNB1: p.Ser33Phe; PIK3CA: p.Lys111Glu; SMARCB1: c.362+1G>C

Supplementary Table 2. Antibodies for western blot

**Antibody**	**Source**	**IDENTIFIER**
Cyclin A2	ABclonal	A19036
Cyclin B1	Proteintech	55004-1-AP
Cyclin D1	Proteintech	60186-1-Ig
Cyclin E1	Proteintech	11554-1-AP
Cyclin E2	ABclonal	A9305
p-CDK1	ABclonal	AP1350
CDK1	ABclonal	A11420
CDK2	Proteintech	60312-1-Ig
CDK4	ABclonal	A0366
CDK6	ABclonal	A0106
p16	ABclonal	A0262
p21	ABclonal	A1483
p27	ABclonal	A0290
c-MYC	CST	5605
p-H2AX	CST	2577
Pro-caspase 3	CST	9662
Cleaved Caspase 3	CST	9661
Pro-caspase 7	CST	12827
Cleaved Caspase 7	CST	8438
Bcl2	ABclonal	A0208
Bax	ABclonal	A19684
Bim	ABclonal	A19702
PARP	CST	9542
MYBL2	Proteintech	18896-1-AP
RRM2	ABclonal	A3424
p-ERK	CST	4370
ERK	CST	4695
p-AKT	CST	4060
AKT	CST	4691
β-Actin	Proteintech	HRP-60008
GAPDH	Proteintech	HRP-60004

Supplementary Table 3. PCR sequences

Gene	Sense	Antisense
MYBL2	CTTGAGCGAGTCCAAAGACTG	AGTTGGTCAGAAGACTTCCCT
RRM2	GCAGCAAGCGATGGCATAGT	GGGCTTCTGTAATCTGAACTTC

Supplementary Table 4. siRNA sequences for knockdown

Gene	siRNA sequence
siNC	UUCUCCGAACGUGUCACGUTT
siMYBL2#1	GCCAUGGACCAAAGAGGAATT
siMYBL2#1	CCGUCCCUCCUACCAUAAATT
siMYBL2#1	CCCAUCGAGUACCAUGAUATT
siMYBL2#1	CGUCGAUAUUCUGGCUCAATT

Supplementary Table 5. Clinical characteristics of patients in TMA

Characteristic	Count / Range [cases (%)]
Total Patients	78
Gender	
Male	46 (59.0)
Female	32 (41.0)
Age (Years)	40 - 82 (Median: 55.5)
40-49 years	12 (15.4)
50-59 years	37 (47.4)
60-69 years	21 (26.9)
≥70 years	8 (10.3)
Clinical Stage (AJCC)	
Stage I	17 (21.7)
Stage II	24 (30.8)
Stage III	26 (33.3)
Stage IV	11 (14.1)
Treatment	
Surgery	57 (73.1)
Chemotherapy	12 (15.4)
Symptomatic Treatment	6 (7.6)
Traditional Chinese Medicine	1 (0.01)
Targeted Therapy	1 (0.01)
Immunotherapy	1 (0.01)
