# Supplementary material for: WEE1 inhibitor exerts synergistic effect with KRAS G12C inhibitor via MYBL2-RRM2 axis in KRASG12C-mutant lung cancer
Source: Cell Death Dis. 2025 Aug 30;16(1):661. doi: 10.1038/s41419-025-07992-4 (PMC12398517; doi:10.1038/s41419-025-07992-4)

Fig 3I

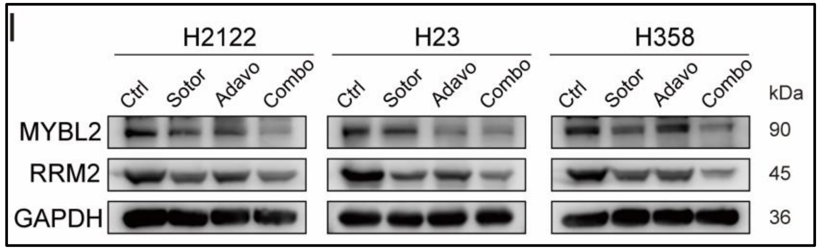
I
H2122
H23
H358
Ctrl
Sotor
Adavo
Combo
kDa
MYBL2
RRM2
GAPDH
90
45
36

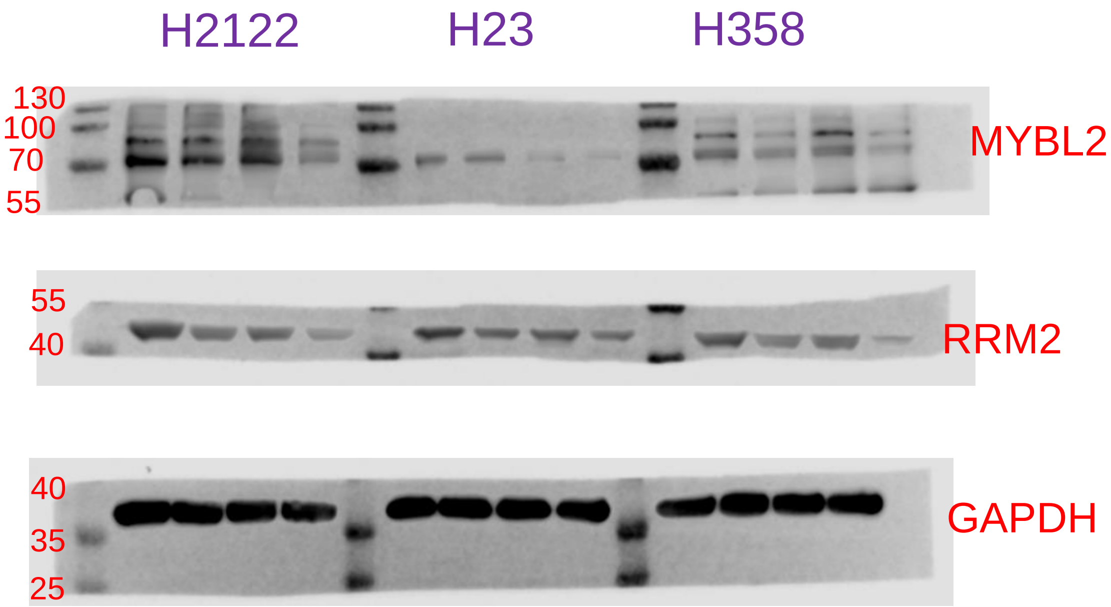
H2122
H23
H358
130
100
70
55
MYBL2
55
40
RRM2
40
35
25
GAPDH

Fig 3J

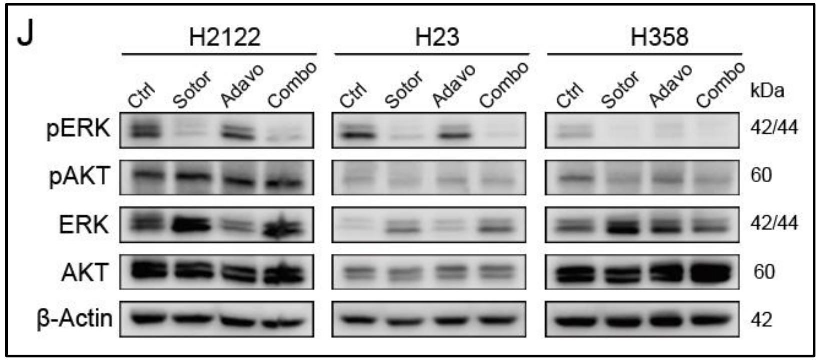
J
H2122
H23
H358
Ctrl
Sotor
Adavo
Combo
kDa
pERK
pAKT
ERK
AKT
β-Actin
42/44
60
42/44
60
42

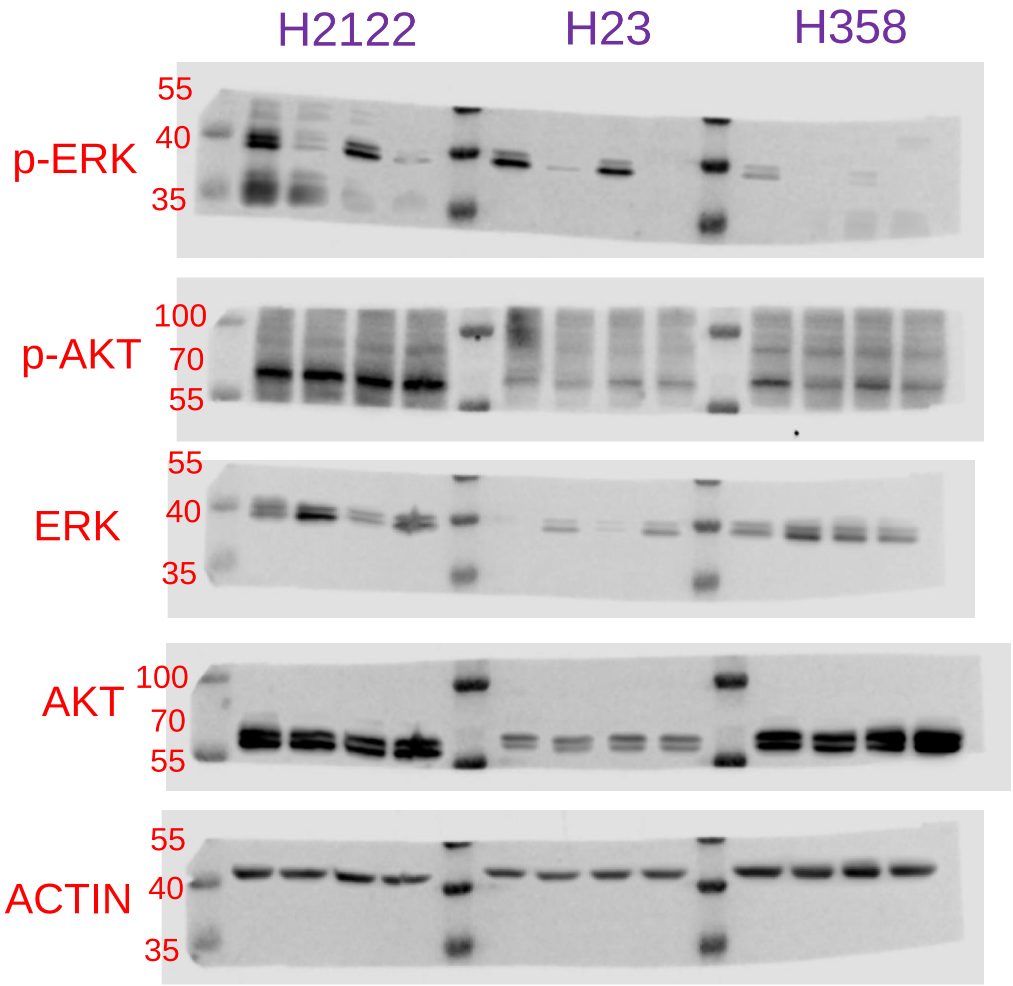
H2122
H23
H358
55
40
35
p-ERK
100
70
55
p-AKT
55
40
35
ERK
100
70
55
AKT
55
40
35
ACTIN

Fig 4G

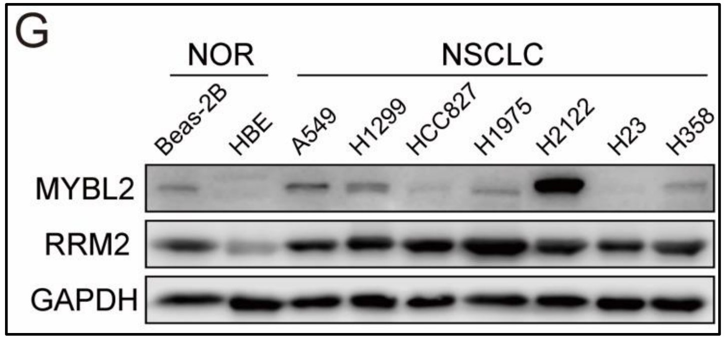

MYBL2

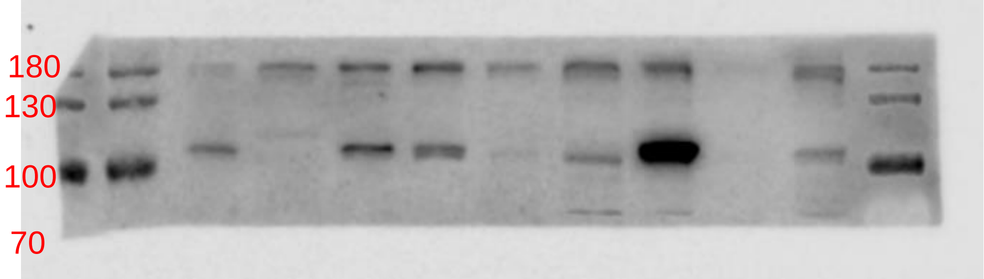

RRM2

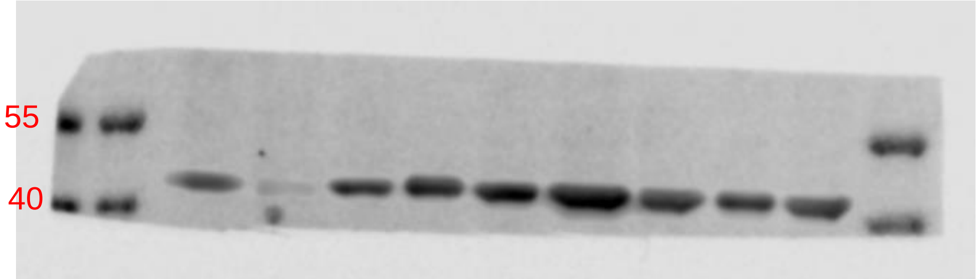

GAPDH

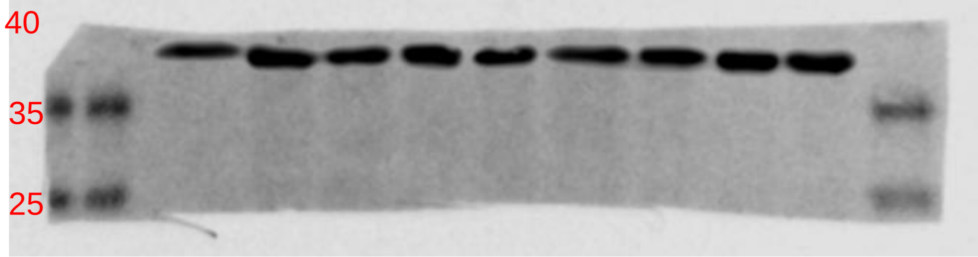

Fig 5A

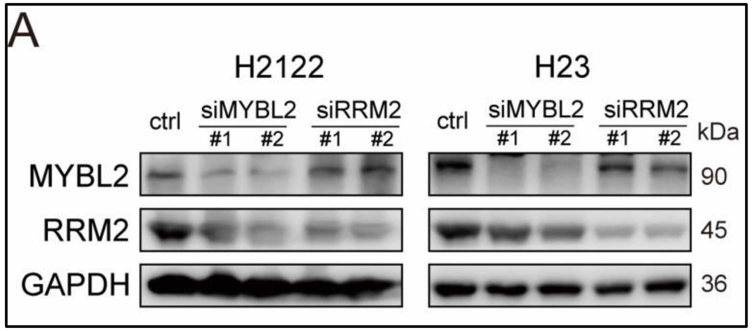
A
H2122
H23
ctrl
siMYBL2
siRRM2
#1
#2
kDa
MYBL2
RRM2
GAPDH
90
45
36

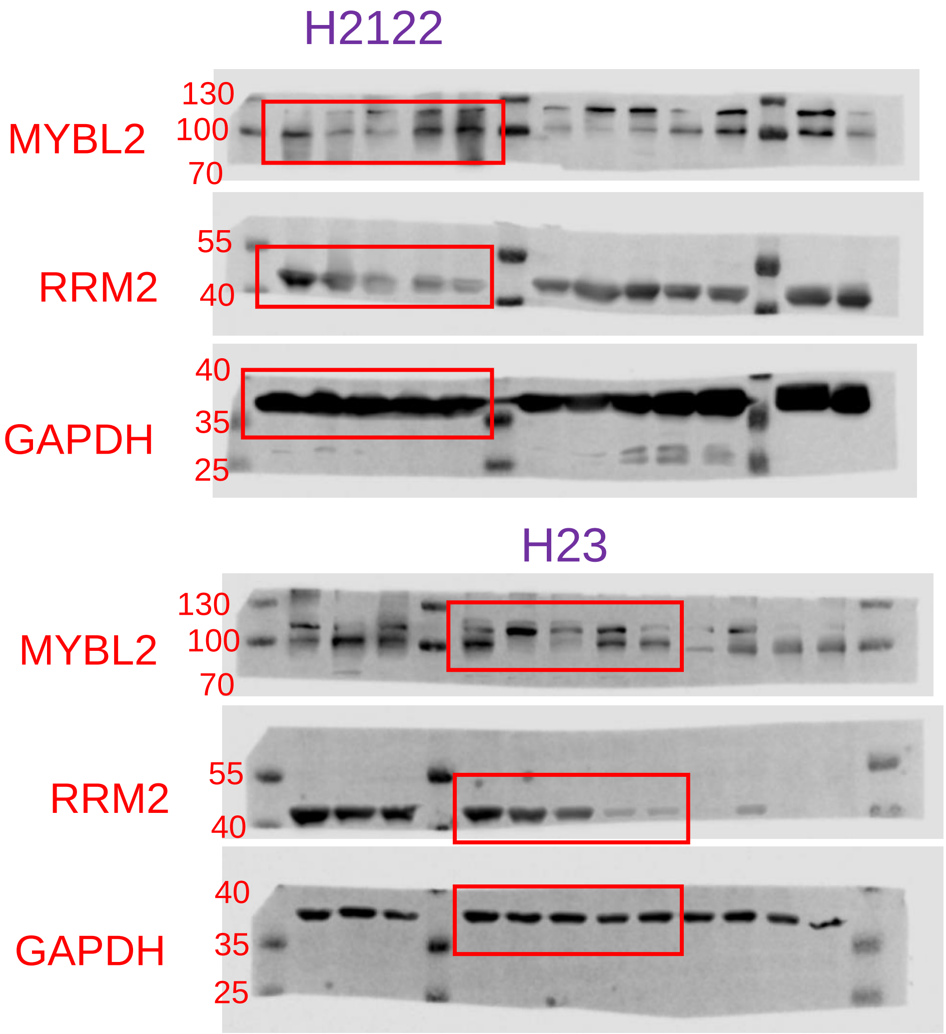
H2122
MYBL2
130
100
70
RRM2
55
40
GAPDH
40
35
25
H23
MYBL2
130
100
70
RRM2
55
40
GAPDH
40
35
25

Fig 5G

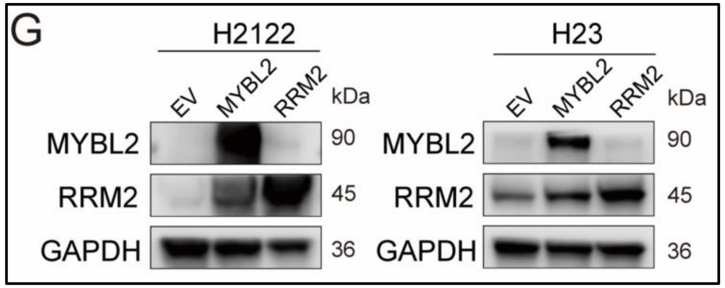
G
H2122
H23
EV
MYBL2
RRM2
kDa
MYBL2
RRM2
GAPDH
90
45
36

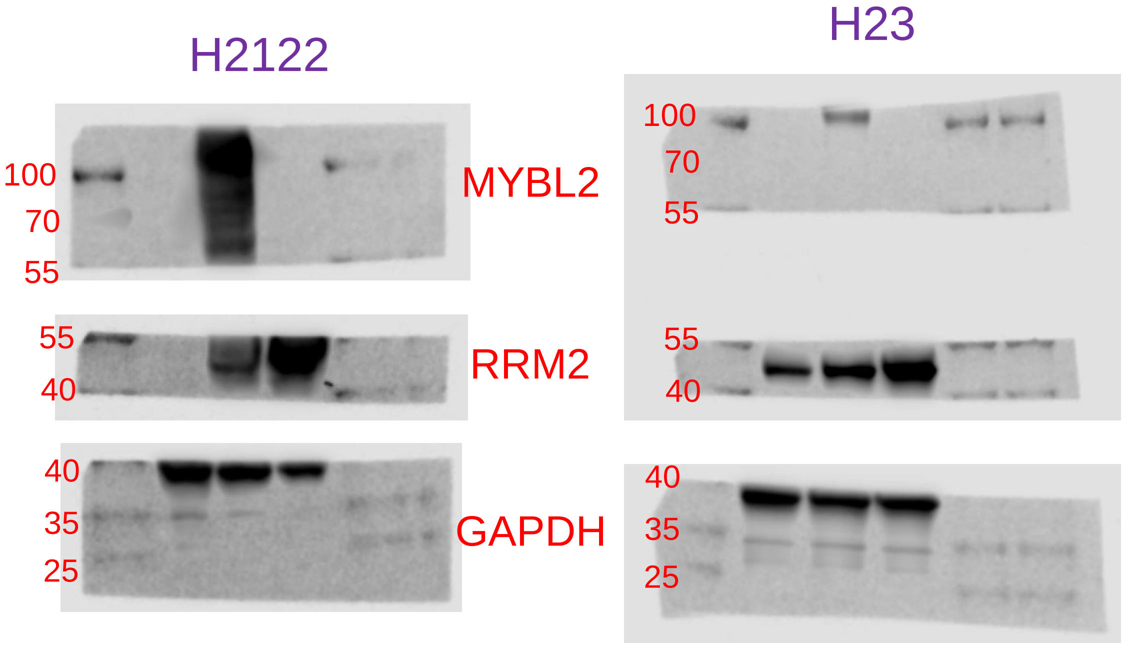
H2122
H23
MYBL2
100
70
55
RRM2
55
40
GAPDH
40
35
25

Fig S2E

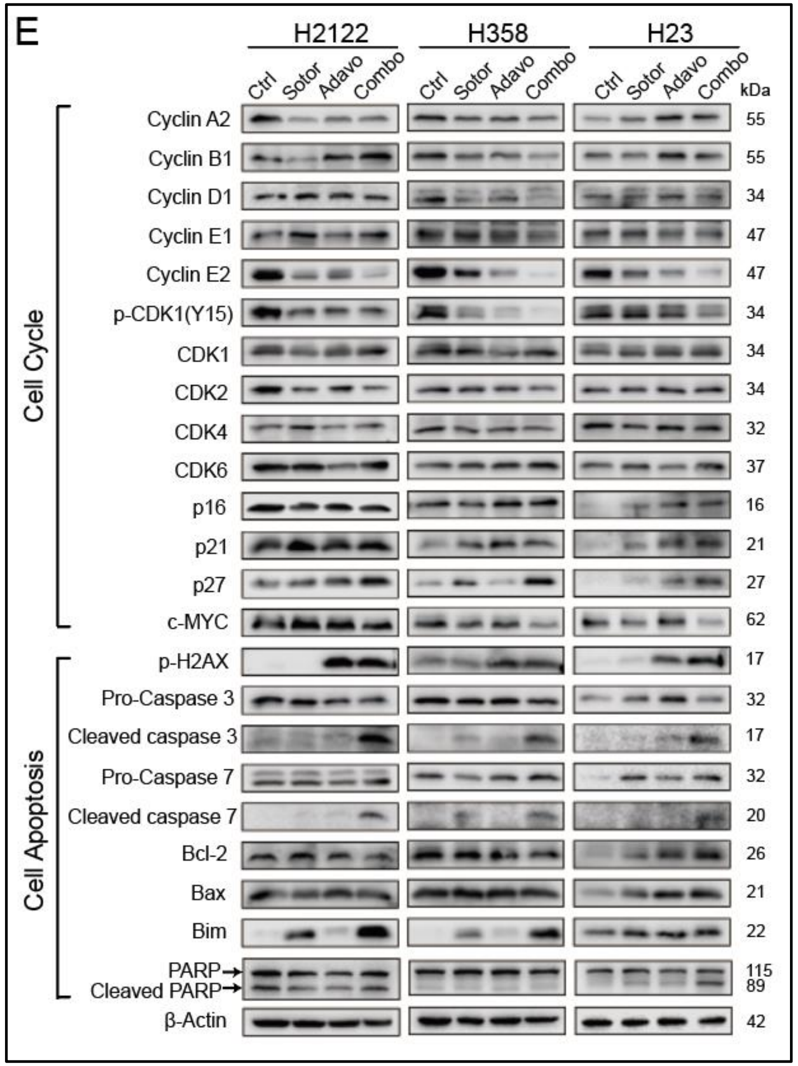

H2122

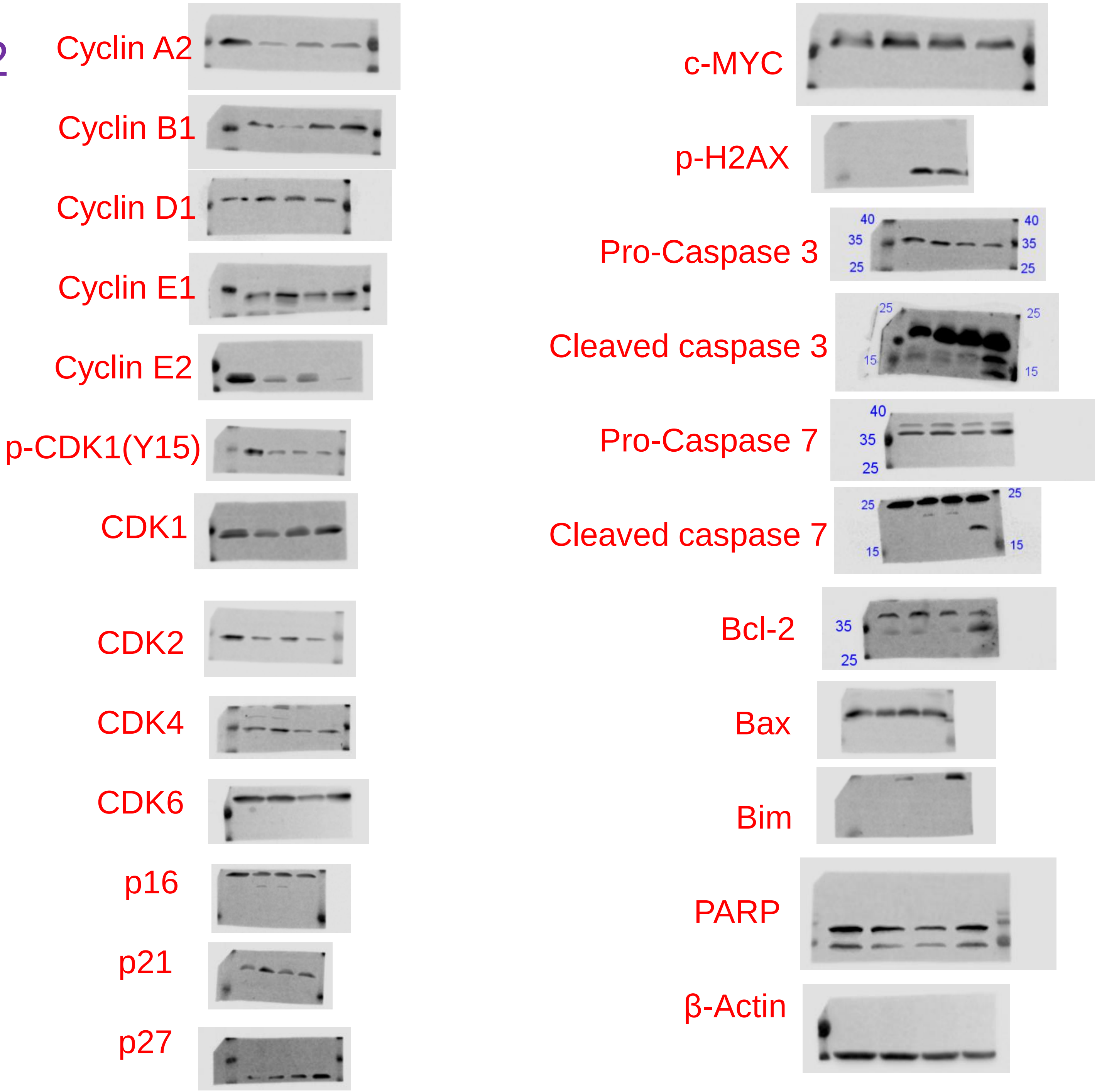

Fig S2E

H358

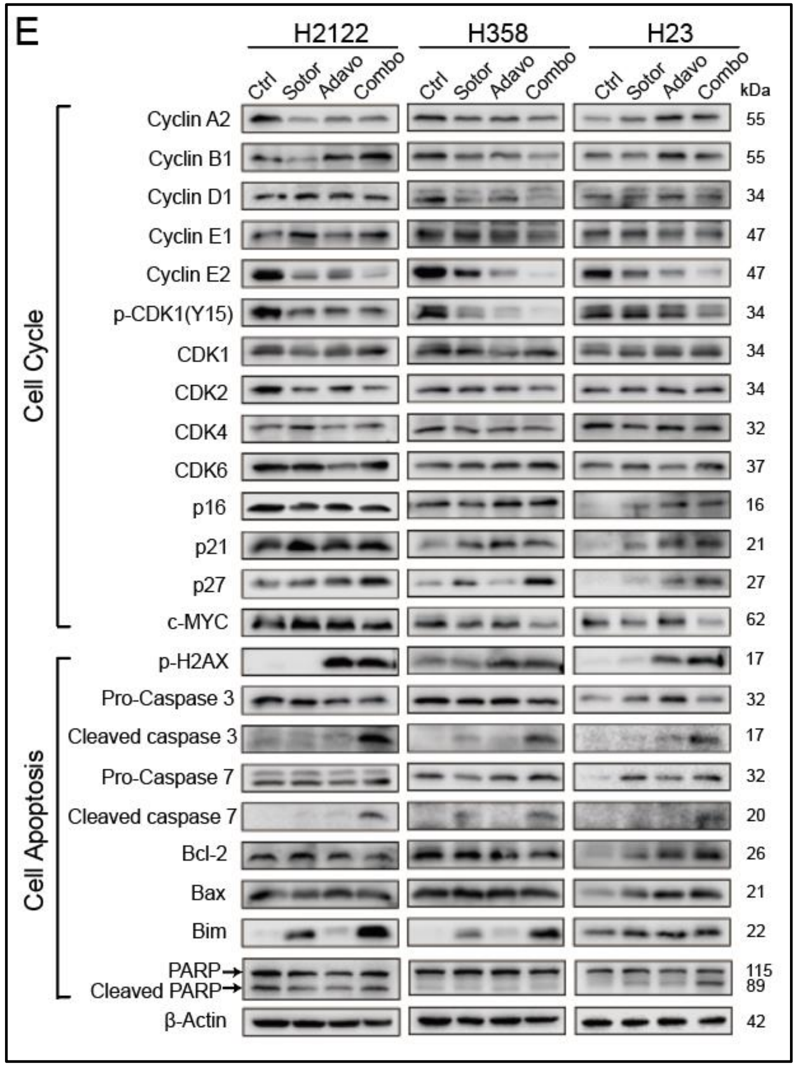
E
H2122
H358
H23
Ctrl
Sotor
Adavo
Combo
kDa
Cell Cycle
Cell Apoptosis
Cyclin A2 55
Cyclin B1 55
Cyclin D1 34
Cyclin E1 47
Cyclin E2 47
p-CDK1(Y15) 34
CDK1 34
CDK2 34
CDK4 32
CDK6 37
p16 16
p21 21
p27 27
c-MYC 62
p-H2AX 17
Pro-Caspase 3 32
Cleaved caspase 3 17
Pro-Caspase 7 32
Cleaved caspase 7 20
Bcl-2 26
Bax 21
Bim 22
PARP 115
Cleaved PARP 89
β-Actin 42

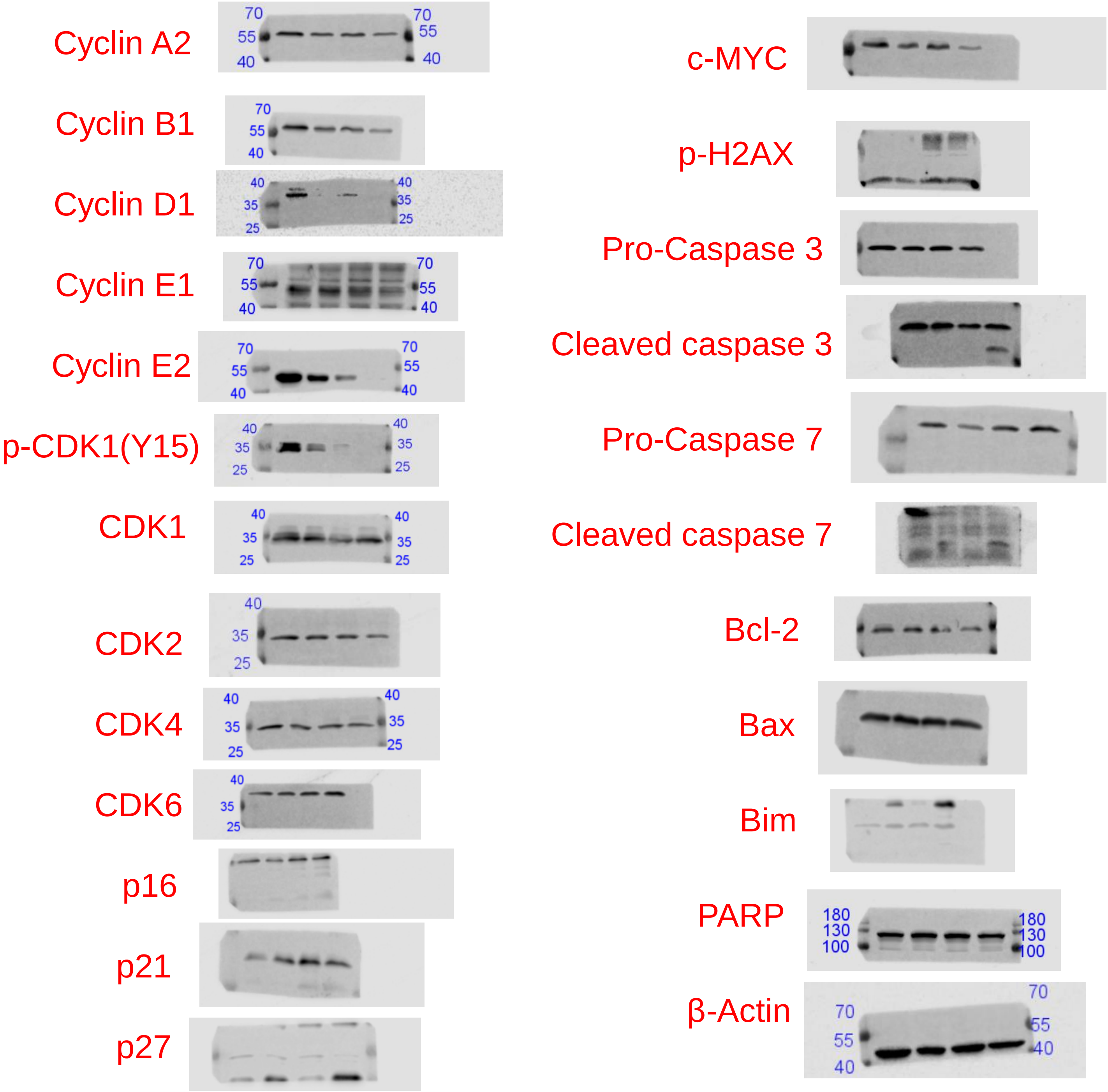
Cyclin A2
Cyclin B1
Cyclin D1
Cyclin E1
Cyclin E2
p-CDK1(Y15)
CDK1
CDK2
CDK4
CDK6
p16
p21
p27
c-MYC
p-H2AX
Pro-Caspase 3
Cleaved caspase 3
Pro-Caspase 7
Cleaved caspase 7
Bcl-2
Bax
Bim
PARP
β-Actin

Fig S2E

H23

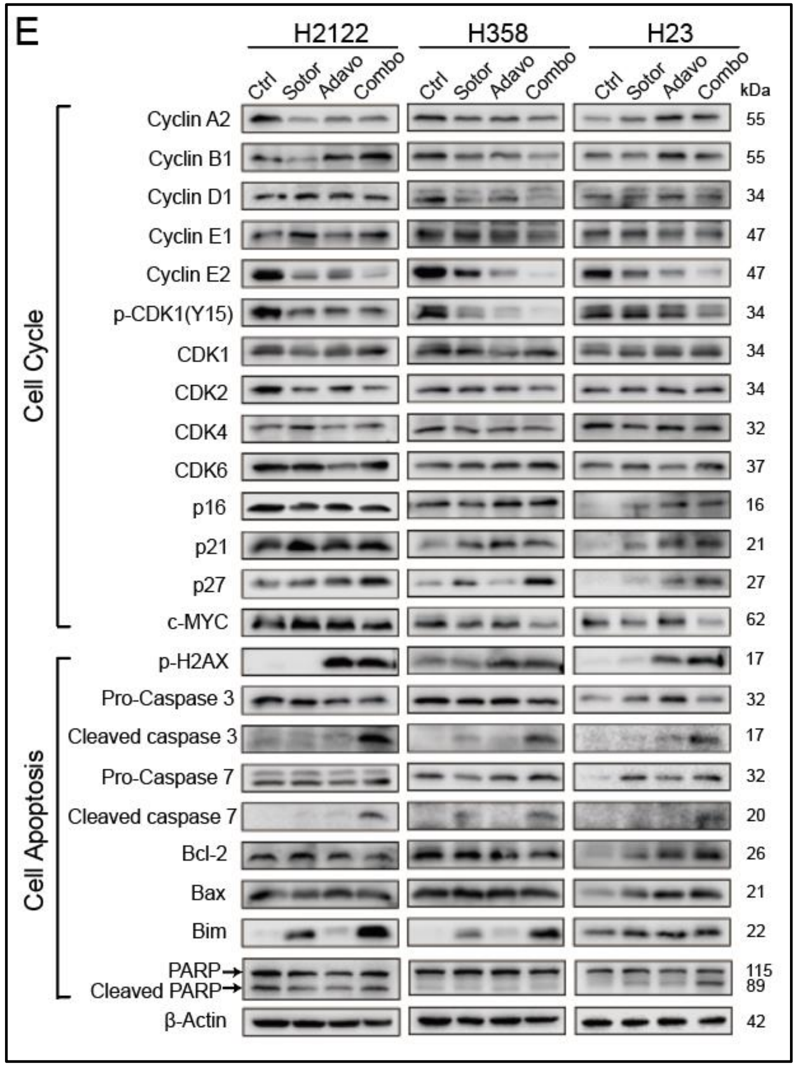
E
H2122
H358
H23
Ctrl
Sotor
Adavo
Combo
kDa
Cyclin A2 55
Cyclin B1 55
Cyclin D1 34
Cyclin E1 47
Cyclin E2 47
p-CDK1(Y15) 34
CDK1 34
CDK2 34
CDK4 32
CDK6 37
p16 16
p21 21
p27 27
c-MYC 62
p-H2AX 17
Pro-Caspase 3 32
Cleaved caspase 3 17
Pro-Caspase 7 32
Cleaved caspase 7 20
Bcl-2 26
Bax 21
Bim 22
PARP 115
Cleaved PARP 89
β-Actin 42
Cell Cycle
Cell Apoptosis

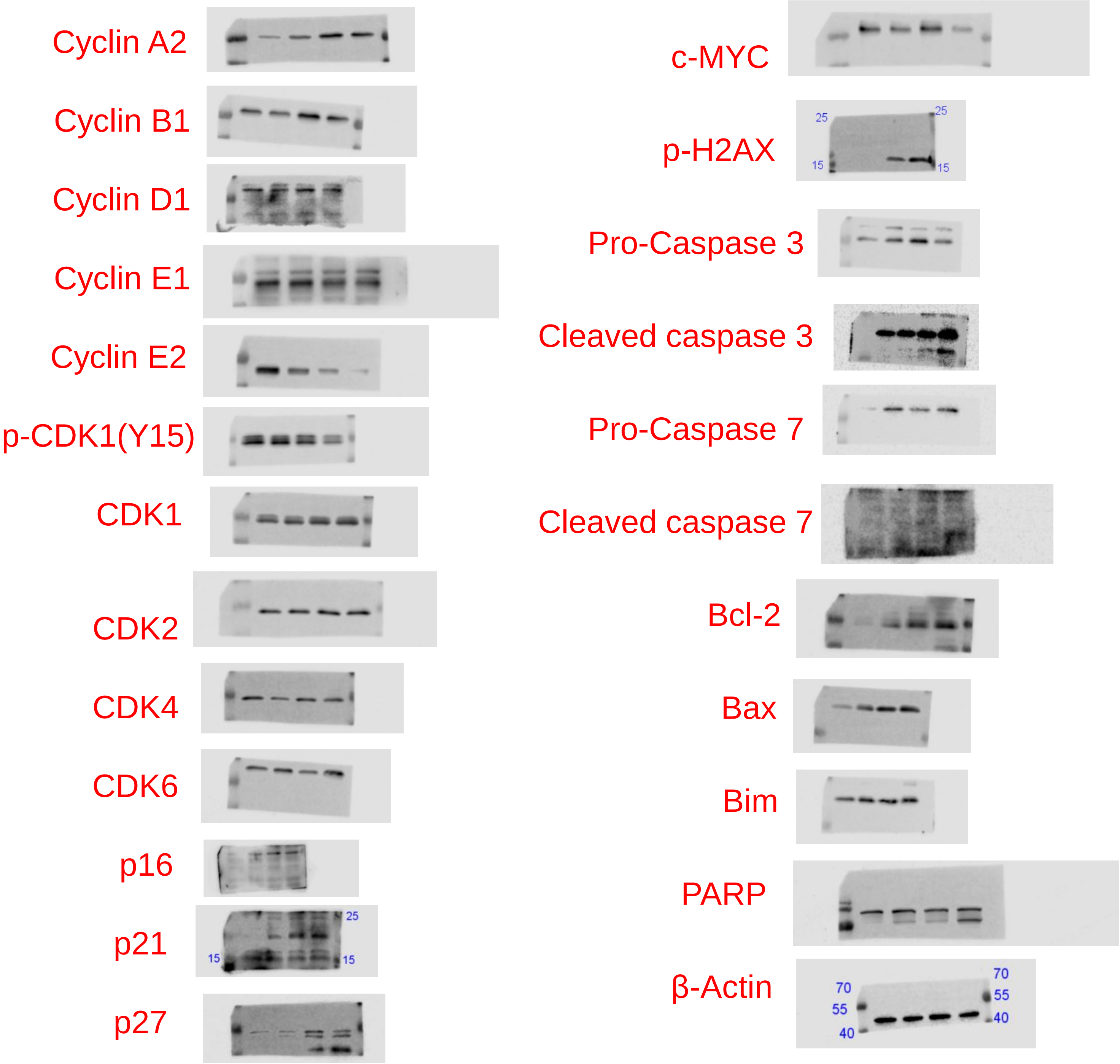
Cyclin A2
Cyclin B1
Cyclin D1
Cyclin E1
Cyclin E2
p-CDK1(Y15)
CDK1
CDK2
CDK4
CDK6
p16
p21
25
15
p27
c-MYC
p-H2AX
25
15
Pro-Caspase 3
Cleaved caspase 3
Pro-Caspase 7
Cleaved caspase 7
Bcl-2
Bax
Bim
PARP
β-Actin
70
55
40

Fig S5G

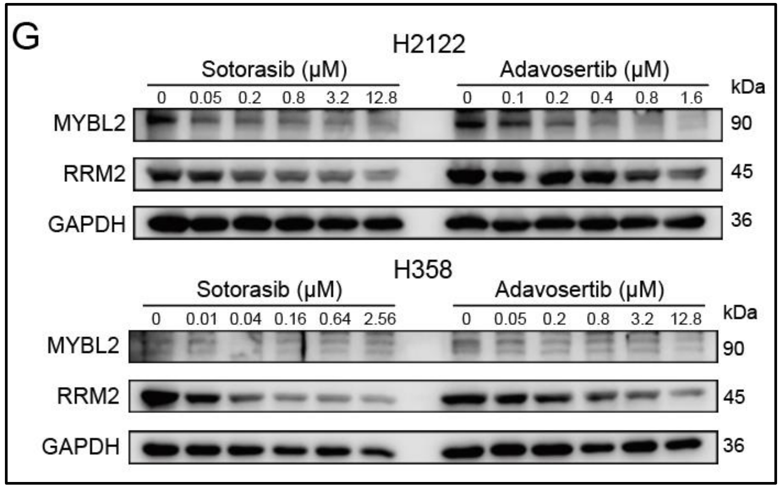
G
H2122
Sotorasib (μM)
Adavosertib (μM)
0 0.05 0.2 0.8 3.2 12.8
0 0.1 0.2 0.4 0.8 1.6
kDa
MYBL2
90
RRM2
45
GAPDH
36
H358
Sotorasib (μM)
Adavosertib (μM)
0 0.01 0.04 0.16 0.64 2.56
0 0.05 0.2 0.8 3.2 12.8
kDa
MYBL2
90
RRM2
45
GAPDH
36

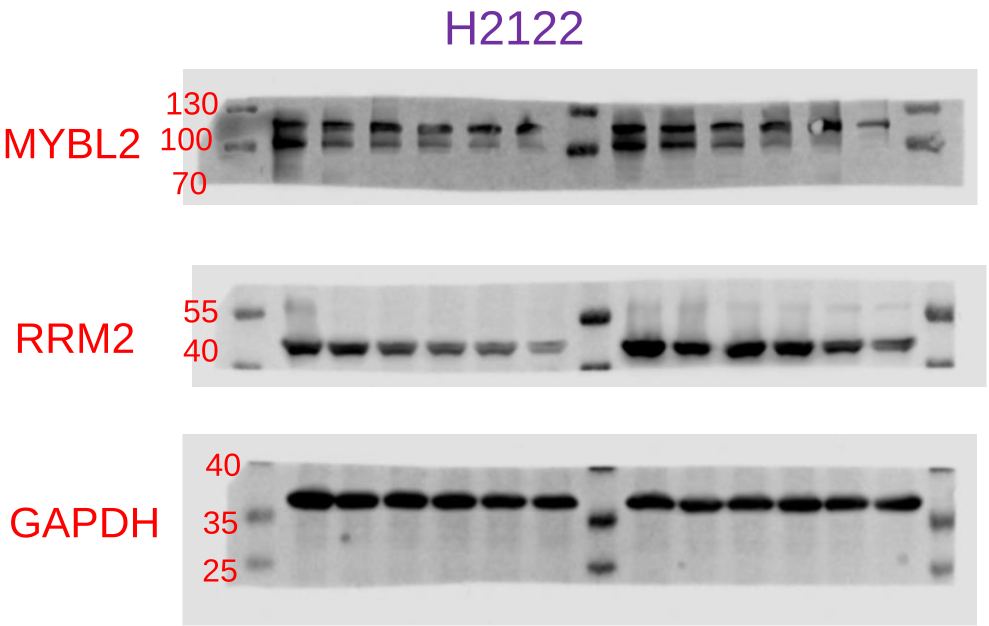
H2122
MYBL2
130
100
70
RRM2
55
40
GAPDH
40
35
25

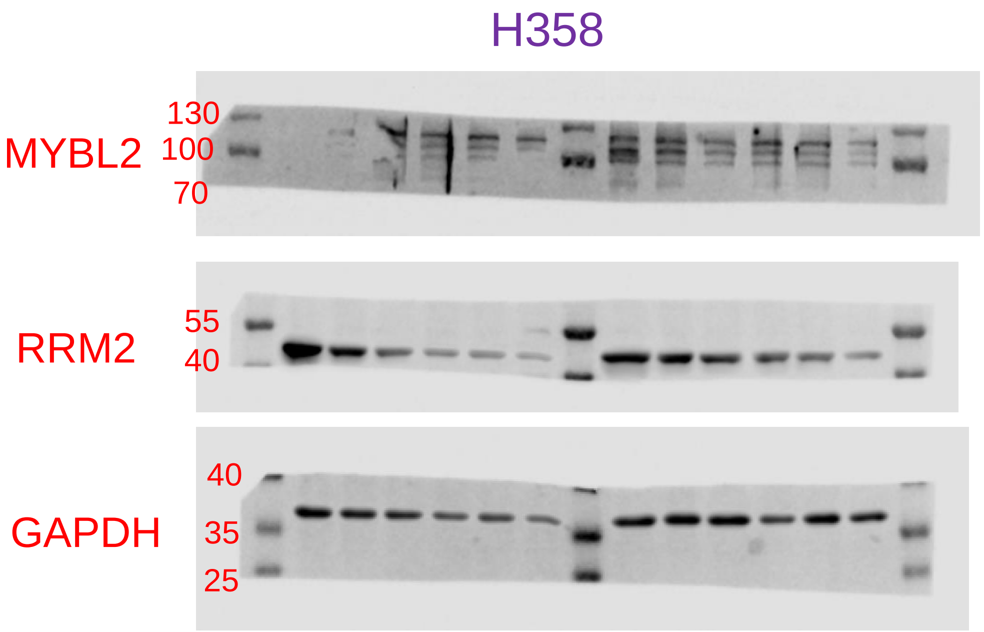
H358
MYBL2
130
100
70
RRM2
55
40
GAPDH
40
35
25

Fig S5H

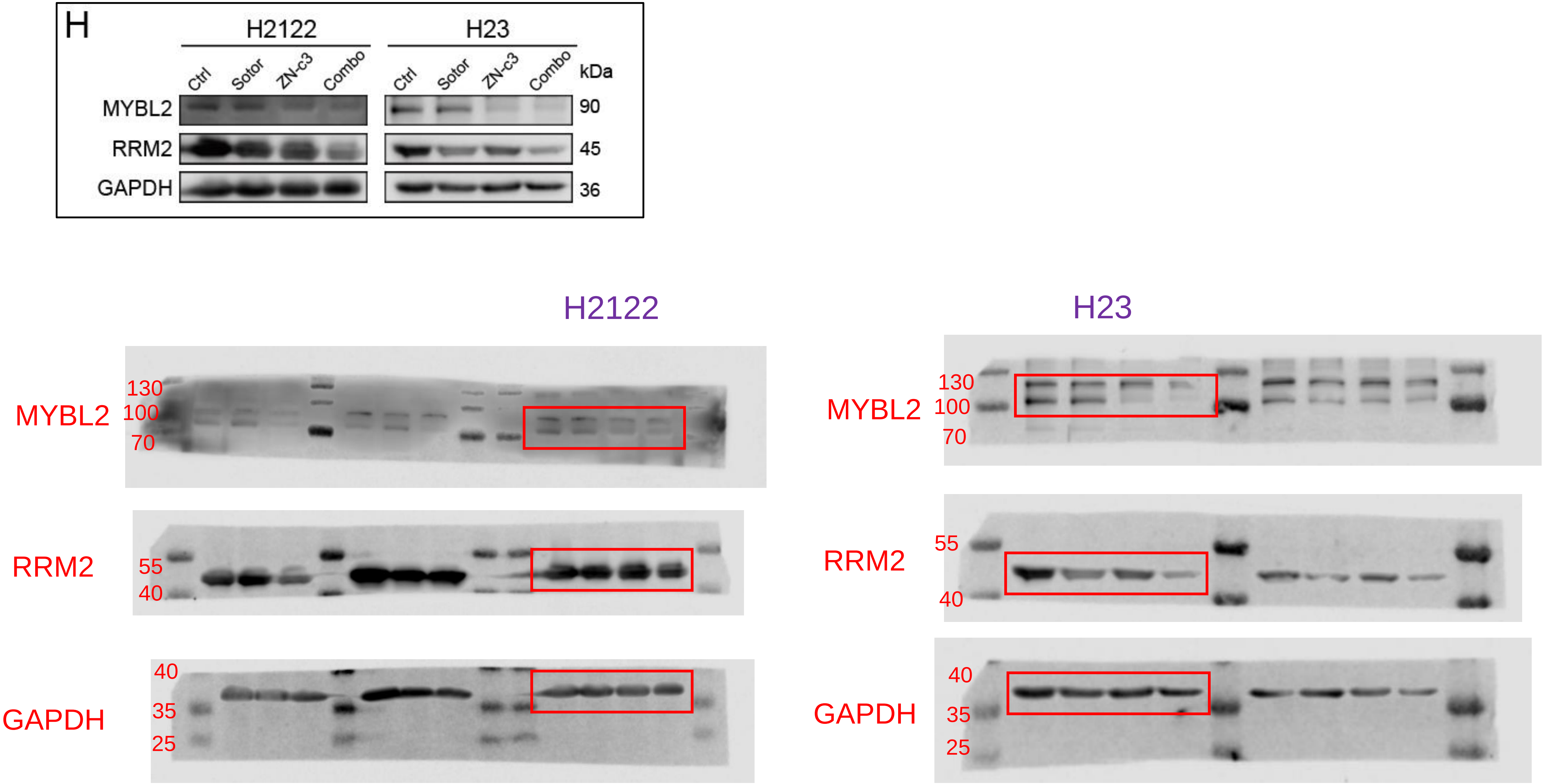
H
H2122
H23
Ctrl
Sotor
ZN-c3
Combo
kDa
MYBL2
RRM2
GAPDH
90
45
36
H2122
H23
MYBL2
130
100
70
RRM2
55
40
GAPDH
40
35
25

Fig S6B

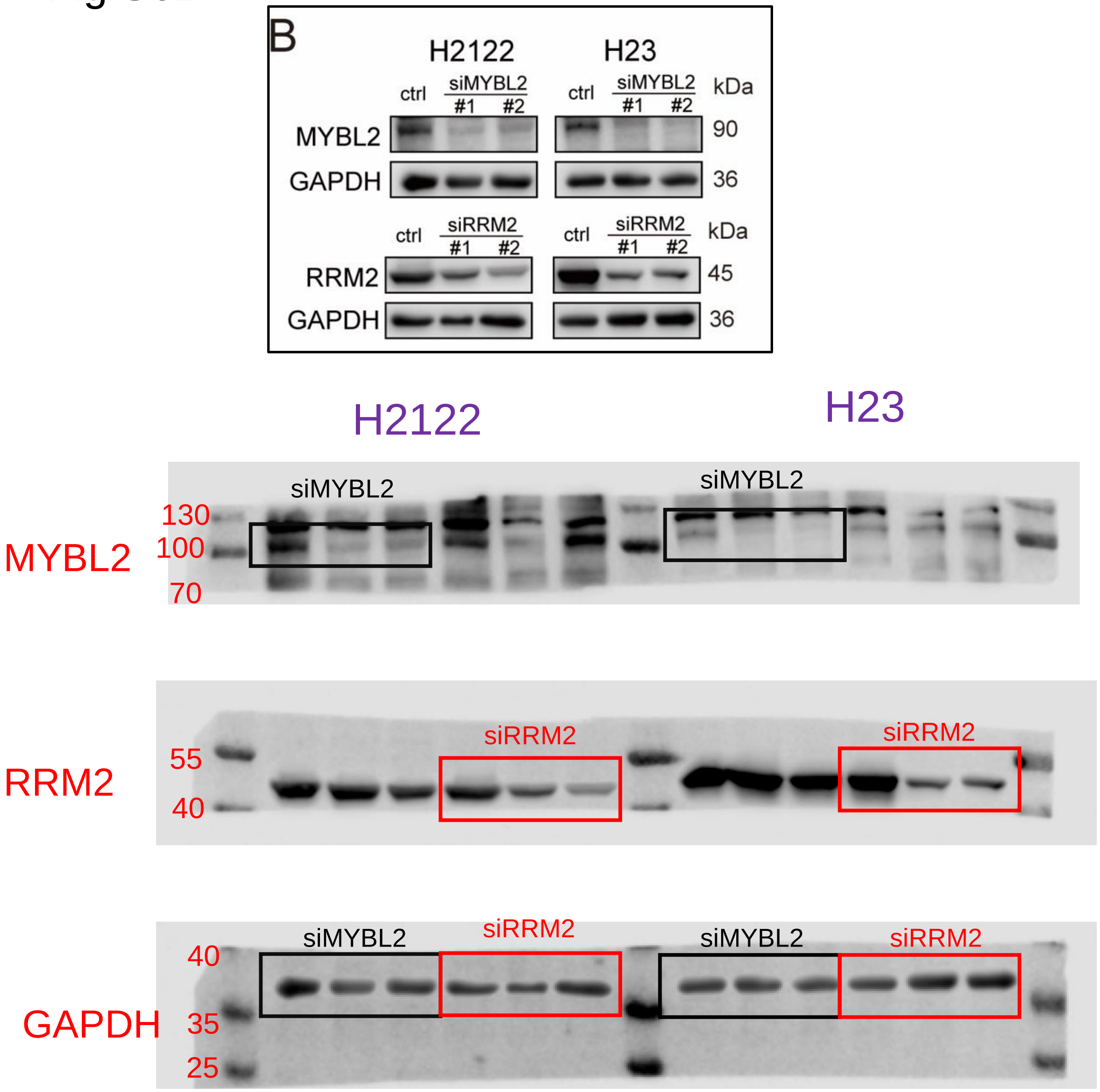

Fig S6F

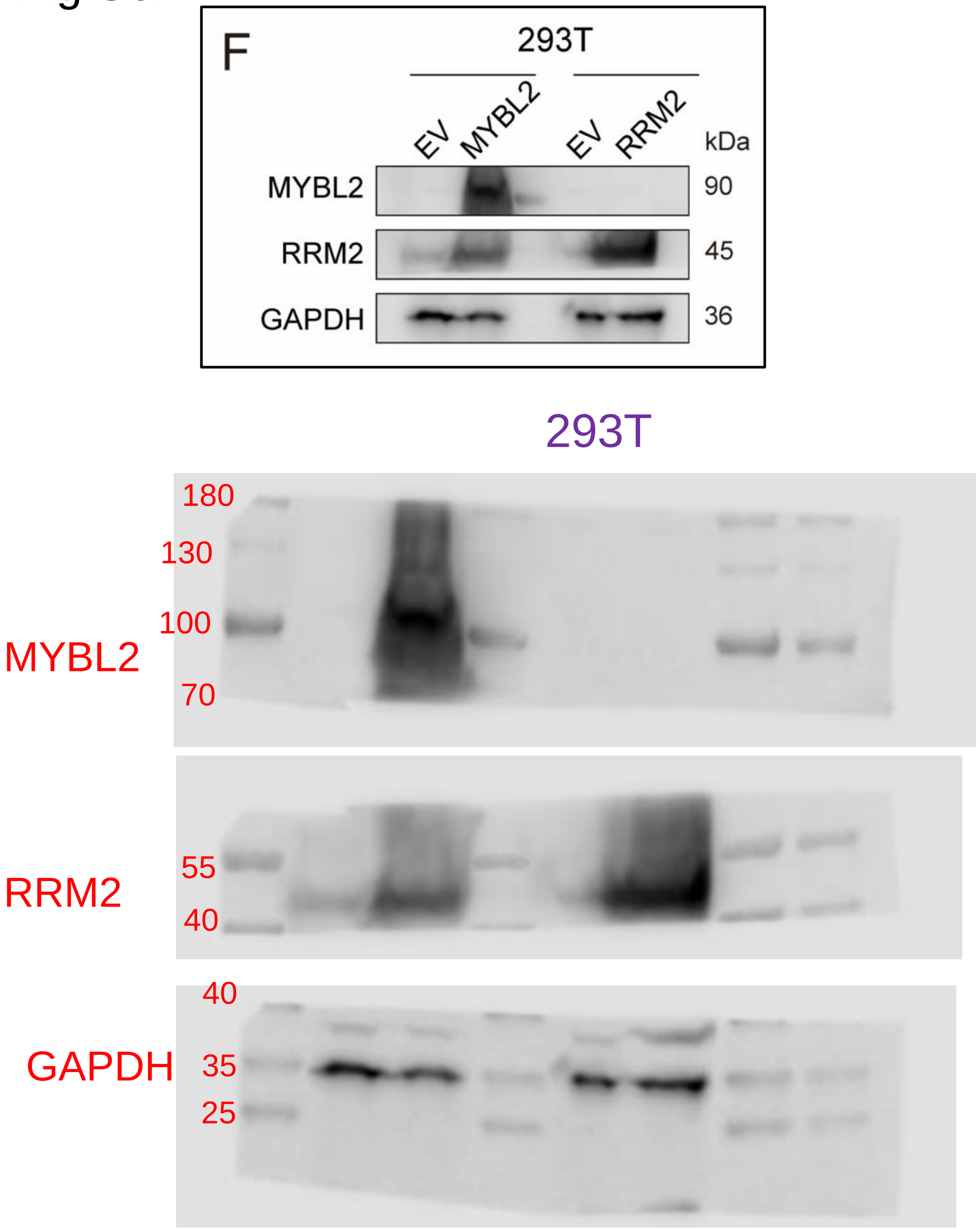

Supplement: Supplementary file 2 — WB raw data [file 41419_2025_7992_MOESM2_ESM.pdf]
